# A yeast-based reverse genetics system to generate HCoV-OC43 reporter viruses encoding an eighth subgenomic RNA

**DOI:** 10.1128/jvi.01671-24

**Published:** 2025-01-30

**Authors:** Brett A. Duguay, Trinity H. Tooley, Eric S. Pringle, John R. Rohde, Craig McCormick

**Affiliations:** 1 Department of Microbiology and Immunology, Dalhousie University 152982 , Halifax, Nova Scotia, Canada; The Ohio State University, Columbus, Ohio, USA

**Keywords:** virus, coronavirus, HCoV-OC43, reverse genetics, yeast, transformation-associated recombination, TAR, mutagenesis, reporter virus

## Abstract

Coronaviruses have large, positive-sense single-stranded RNA genomes that challenge conventional strategies for mutagenesis. Yeast genetics has been used to manipulate large viral genomes, including those of herpesviruses and coronaviruses. This method, known as transformation-associated recombination (TAR), involves assembling complete viral genomes from dsDNA copies of viral genome fragments via homologous recombination in *Saccharomyces cerevisiae*. Here, we report our development of a TAR assembly and mutagenesis system for the endemic, seasonal human coronavirus (HCoV) strain OC43. HCoV-OC43 generally causes mild respiratory symptoms and is classified as a biosafety level 2 agent, making it useful for studying fundamental aspects of coronavirus biology and for comparative studies of more highly pathogenic betacoronaviruses. Following cDNA synthesis from HCoV-OC43 viral RNA, we generated five plasmids encompassing ~7.2 kb portions of the *ORF1ab* gene, the *NS2* to *M* segment, or the *N* gene and structured to facilitate reporter gene insertions in the *M*-to-*N* intergenic region. Using these plasmids, we completed independent assemblies of yeast centromeric plasmids encoding *ORF1ab*, *NS2a* to *N*, as well as full-length HCoV-OC43 plasmids. A wild-type virus (OC43^YA^), as well as mClover3-H2B (OC43-mClo^YA^), mRuby3-H2B (OC43-mRuby^YA^), and mCardinal (OC43-mCard^YA^) reporter viruses, were rescued. The OC43-mClo^YA^ reporter virus replicated comparably to an OC43 reference strain and produced the mClover3-H2B protein from a novel subgenomic RNA through insertion of an eighth body transcription regulatory sequence, preventing the need to delete or mutate viral genes. This updated HCoV-OC43 reverse genetics system will contribute to a better understanding of betacoronavirus host-pathogen interactions and can accelerate studies of novel antivirals.

**IMPORTANCE:**

Coronaviruses are ubiquitous pathogens that infect humans resulting in both mild and severe respiratory infections. Human coronavirus strain OC43 (HCoV-OC43) is one of many viruses responsible for common colds and is a useful model of more severe coronavirus infections. In this study, we describe an updated HCoV-OC43 mutagenesis system that uses yeast to capture six DNA fragments of the viral RNA genome and assemble them into full-length genomes in yeast/bacterial plasmids. The design of this system allowed for the rapid assembly and rescue of functional HCoV-OC43 viruses, including fluorescent reporter viruses with expanded genetic capacity. This updated reverse genetics system will enhance our ability to monitor viral replication, through building new reporter viruses, while also enhancing the study of betacoronavirus biology through the generation of mutant HCoV-OC43 viruses.

## INTRODUCTION

**Fig 1:**
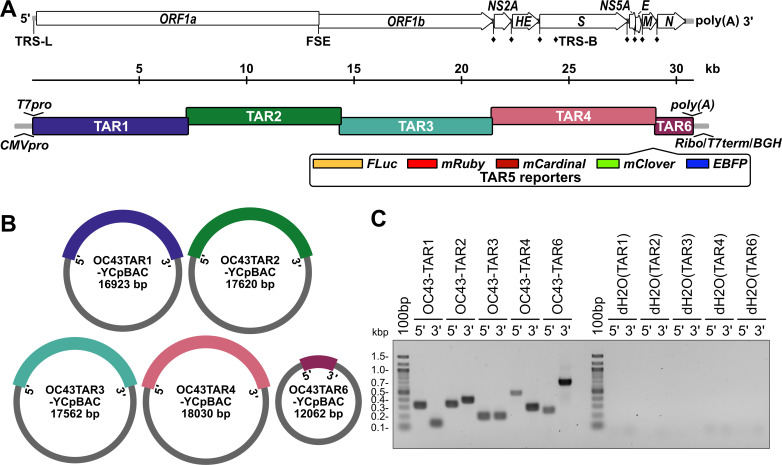
Design of HCoV-OC43 TAR plasmids for yeast assembly of viral sequences. (**A**) A schematic of the HCoV-OC43 genome including annotation of the leader transcription regulatory sequence (TRS-L), ribosomal frameshifting RNA element (FSE), and body TRSs (TRS-B, ♦) (top) and the orientations of the six, overlapping coronavirus TAR fragments (bottom). Inserted regulatory sequences are indicated at the 5′ (*T7pro*, T7 polymerase promoter; *CMVpro*, cytomegalovirus promoter) and 3′ (*poly(A*) tail; *Ribo*, hepatitis delta virus ribozyme; *T7term*, T7 terminator; *BGH*, bovine growth hormone polyadenylation signal) ends of the genome. Five different optional reporter genes (TAR5 reporters) are designed to be inserted between the *M* and *N* genes. (**B**) Illustration of the circular yeast centromeric plasmid/bacterial artificial chromosome (YCpBAC) vectors generated through homologous recombination in yeast with the corresponding HCoV-OC43 TAR sequences. HCoV-OC43 sequences are color-coded to match the diagram in panel A. YCpBAC sequences are shown in gray. (**C**) Plasmids containing the indicated HCoV-OC43 TAR fragments were subjected to PCR using primer sets designed to amplify the 5′ or 3′ junctions between the HCoV-OC43 TAR fragments and the YCpBAC sequences. PCR water controls (dH2O) are shown to the right of the panel. Primer pairs used for screening are listed in Table S2.

Coronaviruses, from the order Nidovirales, are positive-sense, single-stranded RNA [(+)ssRNA] viruses that possess some of the largest (12–41 kb) monopartite RNA genomes and utilize sophisticated strategies to maintain high coding capacity and facilitate replication of their large genomes (1–3). These viruses encode a large polyprotein from *ORF1ab* that encompasses the first two-thirds of their genomes, which is translated using ribosomal frameshifting RNA elements (FSEs) into polyproteins pp1a and pp1ab (Fig. 1A and [4]). These polyproteins are proteolytically processed by the viral papain-like protease (PLpro) and the main protease (Mpro) into 16 non-structural proteins (Nsps) with roles in host subversion, replication compartment formation, and viral RNA production and modification (5). The remaining 3′ third of coronavirus genomes encode accessory and structural proteins. These proteins are translated from a nested set of subgenomic mRNAs (sg-mRNAs) produced through discontinuous transcription utilizing transcription regulatory sequences (TRSs). One TRS is positioned at the 5′ end of the genome (TRS-L), while multiple body-TRSs (TRS-B) are located 5′ of each sg-mRNA translation initiation site (Fig. 1A). These regulatory sequences direct template switching by the viral RNA-dependent RNA polymerase during negative-sense subgenomic RNA ((-)sgRNA) transcription to produce templates for subsequent viral sg-mRNA synthesis (6–8).

Understanding these complex replication strategies of coronaviruses was made possible through the development of reverse genetics systems. These systems work on the premise of regenerating an infectious genomic RNA (gRNA) from a plasmid assembled using complementary DNA (cDNA) fragments. Due to the challenge of working with such large genomes, multiple strategies have been adopted to reassemble full-length coronavirus genomic sequences including RNA recombination (9–11), restriction digestion and *in vitro* ligation (12–32), lambda Red-mediated recombination in bacteria (33, 34), Gibson assembly (35), circular polymerase extension reaction (CPER) (36–40), Golden Gate assembly (41), DNA fragment recombination in mammalian cells (42, 43), and transformation-associated recombination (TAR) in yeast (44–46).

Based on our experience using the human coronavirus OC43 (HCoV-OC43) as a biosafety level 2 model of betacoronavirus infection, we set out to develop a yeast-based mutagenesis platform of HCoV-OC43 to support antiviral drug discovery and host-pathogen interaction research. The TAR method uses the highly efficient *Saccharomyces cerevisiae* homologous recombination system to join numerous transformed DNA fragments with ≥40 bp of DNA homology into yeast centromeric plasmids (YCp) (47). It has been recently applied to the successful assembly of large DNA viruses including herpes simplex virus type 1, human cytomegalovirus (HCMV), Kaposi’s sarcoma-associated herpesvirus (48–50) and to generate cDNA copies of RNA viruses such as swine enteric alphacoronavirus, mouse hepatitis virus (MHV), Middle East respiratory syndrome coronavirus, severe acute respiratory syndrome coronavirus 2 (SARS-CoV-2), Japanese encephalitis virus, porcine epidemic diarrhea virus, and feline coronavirus (44–46, 51, 52).

Prior to beginning our work on HCoV-OC43, only one bacterial artificial chromosome (BAC)-based reverse genetic platform for this virus had been developed (18). Earlier this year, two additional HCoV-OC43 mutagenesis systems were published, both of which inserted reporter sequences within viral open reading frames (32, 40). Here, we describe our work applying synthetic biology techniques and yeast genetics to develop an HCoV-OC43 reverse genetics system culminating in the generation of yeast-assembled wild-type (OC43^YA^), mClover3-H2B reporter (OC43-mClo^YA^), mRuby3-H2B reporter (OC43-mRuby^YA^), and mCardinal reporter (OC43-mCard^YA^) viruses. The viruses we assembled and rescued replicated comparably to a wild-type strain of HCoV-OC43. Importantly, our updated reporter virus platform did not require the mutation of any viral protein. By expressing the mClover3 variant of GFP or mRuby3 variant of RFP (53) fused to histone H2B, these reporters provide improved brightness and exclusively nuclear localization compared to EGFP or RFP, making them well suited for live cell imaging, immunofluorescence, automated image analysis, and flow cytometric analyses of viral replication. Using this system, we have made plasmids containing individual cDNA fragments or partially assembled cDNAs of the viral genome to provide some modularity to the assembly process and to maximize flexibility for performing mutagenesis. Generation of the OC43 reporter viruses demonstrates that HCoV-OC43 tolerates up to a 1246 base insertion in the intergenic region between the *M* and *N* genes and the addition of an eighth TRS-B. Our reporter viruses are the first examples of GFP/RFP-based HCoV-OC43 reporter viruses built without the deletion or mutation of viral coding sequences.

## RESULTS

Viral reverse genetics systems have greatly impacted the understanding of viral biology and can accelerate the study of antiviral interventions by producing reporter viruses to rapidly track infectivity. The first BAC-based, HCoV-OC43 reverse genetics system was developed using overlap PCR products and restriction cloning (18), which led to the creation of luciferase reporter viruses deficient in *NS2A* or *NS5A* (54). More recently, HCoV-OC43 reverse genetics systems using *in vitro* ligation or CPER were used to generate Δ*NS2A::nanoluciferase* or *Orf1b*-*EGFP* in-frame fusion reporter viruses, respectively (32, 40). Previously, work with another betacoronavirus, MHV, demonstrated the ability to insert luciferase genes preceded by independent TRSs to drive reporter gene expression, where reporter gene insertions proximal to the 3′ ends of the viral genome resulted in the most robust expression (10). We reasoned that HCoV-OC43 viruses encoding fluorescent protein reporter genes could be generated using a similar mutagenesis strategy. By introducing an additional TRS into the viral genome, this strategy eliminates the need for fusion proteins or the deletion of viral accessory genes in recombinant HCoV-OC43 strains.

### Design, capture, and assembly of HCoV-OC43 cDNA plasmids

We developed an updated reverse genetics system for HCoV-OC43 that employs efficient homologous recombination in *S. cerevisiae* to assemble viral cDNA fragments. This method of TAR cloning has been applied to cloning complete human genes, yeast chromosomes, and to the assembly of entire bacterial genomes through accurate, stable, recombination-mediated DNA assembly (55–58). Our reverse genetics strategy divided the viral genome into five fragments: Three ~ 7.2 kb portions of the *ORF1ab* genes (TAR1, 2, and 3), a fourth 7.4 kb portion encoding the *NS2* to *M* genes (TAR4), and a short fifth fragment (1.6 kb) containing the *N* gene (TAR6) (Fig. 1A).

We employed two strategies for the rescue of infectious HCoV-OC43, T7 RNA polymerase-directed gRNA production or CMV promoter-driven *in vivo* rescue, which required the insertion of T7 RNA polymerase promoter (*T7pro*) or CMV/RNA polymerase II promoter (*CMVpro*) sequences directly 5′ to the HCoV-OC43 5′ untranslated region (5′UTR) in TAR1 (Fig. 1A). Likewise, essential regulatory sequences were inserted downstream of *N*, including a synthetic stretch of 34 adenosine monophosphates (*poly(A*)) for RNA stability and a hepatitis delta virus ribozyme self-cleaving RNA sequence (*Ribo* [59, 60]) to generate an authentic gRNA 3′ end (Fig. 1A). Downstream of the ribozyme sequence were a T7 RNA polymerase terminator (*T7term*), to use with the *T7pro* system, and a bovine growth hormone polyadenylation signal (*BGH*), for use with the *CMVpro* system (Fig. 1A). First, HCoV-OC43 gRNA was converted to cDNA and PCR amplified. These PCR amplicons were then co-transformed into yeast along with a linearized yeast centromeric plasmid/bacterial artificial chromosome (YCpBAC) plasmid containing unique 5′ and 3′ ends homologous to the various HCoV-OC43 TAR fragments (Fig. 1B; Table S1). The resulting yeast clones were screened by PCR for the 5′ and 3′ DNA junctions between the HCoV-OC43 and YCpBAC sequences and yielded banding patterns indicative of correct fragment capture into the YCpBACs (Fig. 1C; Table S2).

**Fig 2:**
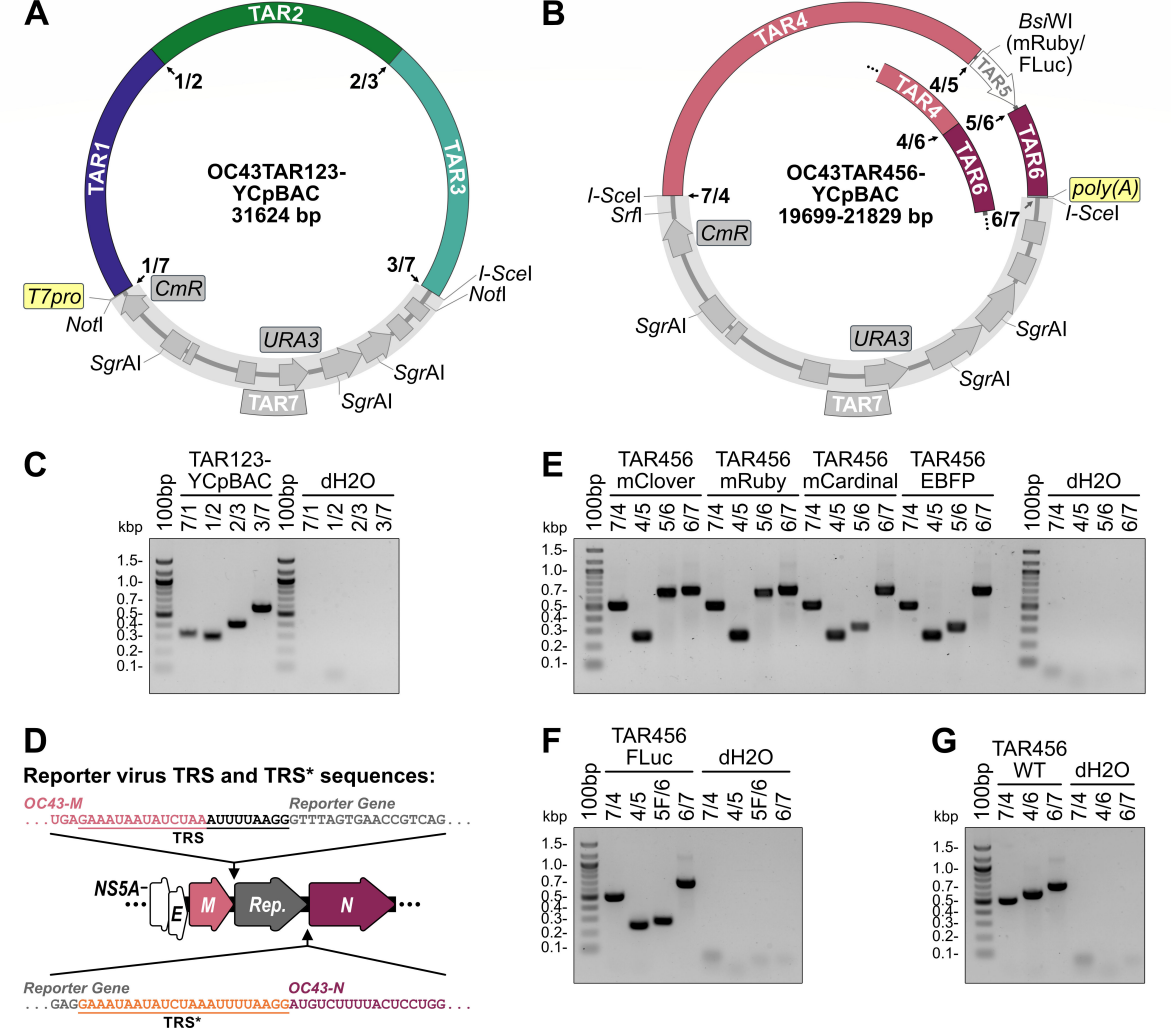
Yeast assembly of plasmids encoding HCoV-OC43 *Orf1ab* and *NS2A-to-N*. Diagrams of the OC43TAR123-YCpBAC plasmid containing HCoV-OC43 TAR fragments 1, 2, and 3 encoding *Orf1ab* (**A**) and OC43TAR456-YCpBACs containing HCoV-OC43 TAR fragments 4, 5 (indicated in white in the plasmid map), and 6 or fragments (for reporter virus assembly) or 4 and 6 (for wild-type (WT) virus assembly; inset) encoding *NS2A* to *N* +/- reporter genes (**B**). HCoV-OC43 sequences are color-coded to match the diagram in Fig. 1. YCpBAC sequences (TAR7) are shown in gray. Numbered junctions detected by PCR (black arrows) and restriction sites used for TAR cloning are indicated. (**C**) Following assembly in yeast, the TAR123-YCpBAC was subjected to PCR using primer sets designed to amplify junctions 7/1, 1/2, 2/3, and 3/7 with water controls (dH2O) to the right of the panel. (**D**) Arrangement of the *M-N* locus in reporter virus genomes with the authentic *N* TRS used to drive reporter gene sgRNA synthesis and an inserted copy of the *N* TRS (TRS*, sequence in orange) for *N* sgRNA synthesis indicated. (**E**) Following assembly in yeast, TAR456-YCpBACs were subjected to PCR using primer sets designed to amplify junctions 7/4, 4/5 (present when flanking a reporter gene), 5/6 (for fluorescence reporter genes), and 6/7 with dH2O controls to the right of the panel. (**F**) PCR screening of assembled TAR456-FLuc-YCpBAC with screening for junctions: 7/4, 4/5, 5F/6 (for the FLuc reporter gene), and 6/7 with dH2O controls to the right of the panel. (**G**) PCR screening of assembled TAR456-WT-YCpBAC with screening for junctions: 7/4, 4/6, and 6/7 with dH2O controls to the right of the panel. Primer pairs used for screening are listed in Table S2. Abbreviations: 100bp, 100 bp ladder; bp/kbp, base/kilobase pairs; *CmR*, chloramphenicol resistance gene; EBFP, enhanced blue fluorescent protein; FLuc, firefly luciferase; *poly(A*), encoded A_34_ sequence; Rep., reporter gene; *T7pro*, T7 polymerase promoter; TRS, transcription regulatory sequence; *URA3*, orotidine 5′-phosphate decarboxylase gene; WT, wild-type.

HCoV-OC43 sequences were reassembled into *Orf1ab* (TAR123) or *NS2A*-to-*N* (TAR456) containing YCpBACs to facilitate subsequent full-length HCoV-OC43 sequence assemblies (Fig. 2A and B). The three TAR plasmids encoding fragments of *Orf1ab* (TAR1, TAR2, and TAR3) were linearized and co-transformed with a YCpBAC (TAR7, in gray) sequence to facilitate assembly of OC43TAR123-YCpBAC in yeast (Fig. 2A). The correct junction amplicons were obtained during screening for the junctions 7/1, 1/2, 2/3, and 3/7 (Fig. 2C; Table S2), indicating the correct assembly of these sequences. Likewise, the three TAR plasmids encoding *NS2A*, *HE*, *S*, *NS5A*, *E*, and *M* (TAR4), the reporter genes *mClover3-H2B* (mClover3 fused to histone H2B), *mRuby3-H2B* (mRuby3 fused to histone H2B), *mCardinal*, *EBFP2*, or firefly *luciferase* (*FLuc*) (TAR5), and *N* (TAR6) were linearized and co-transformed into yeast with a YCpBAC (TAR7, in gray) to assemble the OC43TAR456-YCpBAC plasmids (Fig. 2B). This design provided the flexibility to insert various reporter gene sequences, driven from the authentic *N* TRS, into the region between *M* and *N*. Since the authentic TRS for *N* now drives reporter gene sgRNA synthesis, the expression of *N* was maintained through the insertion of a duplicated 23 base TRS-containing element upstream of the *N* start codon (TRS*, Fig. 2D). The correct junction amplicons were obtained during screening for the junctions 7/4, 4/5, 5/6, 5F/6 (FLuc only), and 6/7 (Fig. 2E and F; Table S2), indicating the correct assembly of the reporter plasmids. Assembly of a wild-type *NS2A-N* sequence lacking a reporter gene was completed using a 297 bp TAR5 PCR amplicon. To provide the flexibility to insert sequences between *M* (TAR4) and *N* (TAR6), these TAR fragments have minimal overlap, thus requiring the short PCR amplicon for assembly. The anticipated DNA junction amplicons (7/4, 4/6, and 6/7) were obtained for this wild-type plasmid (Fig. 2G; Table S2).

**Fig 3:**
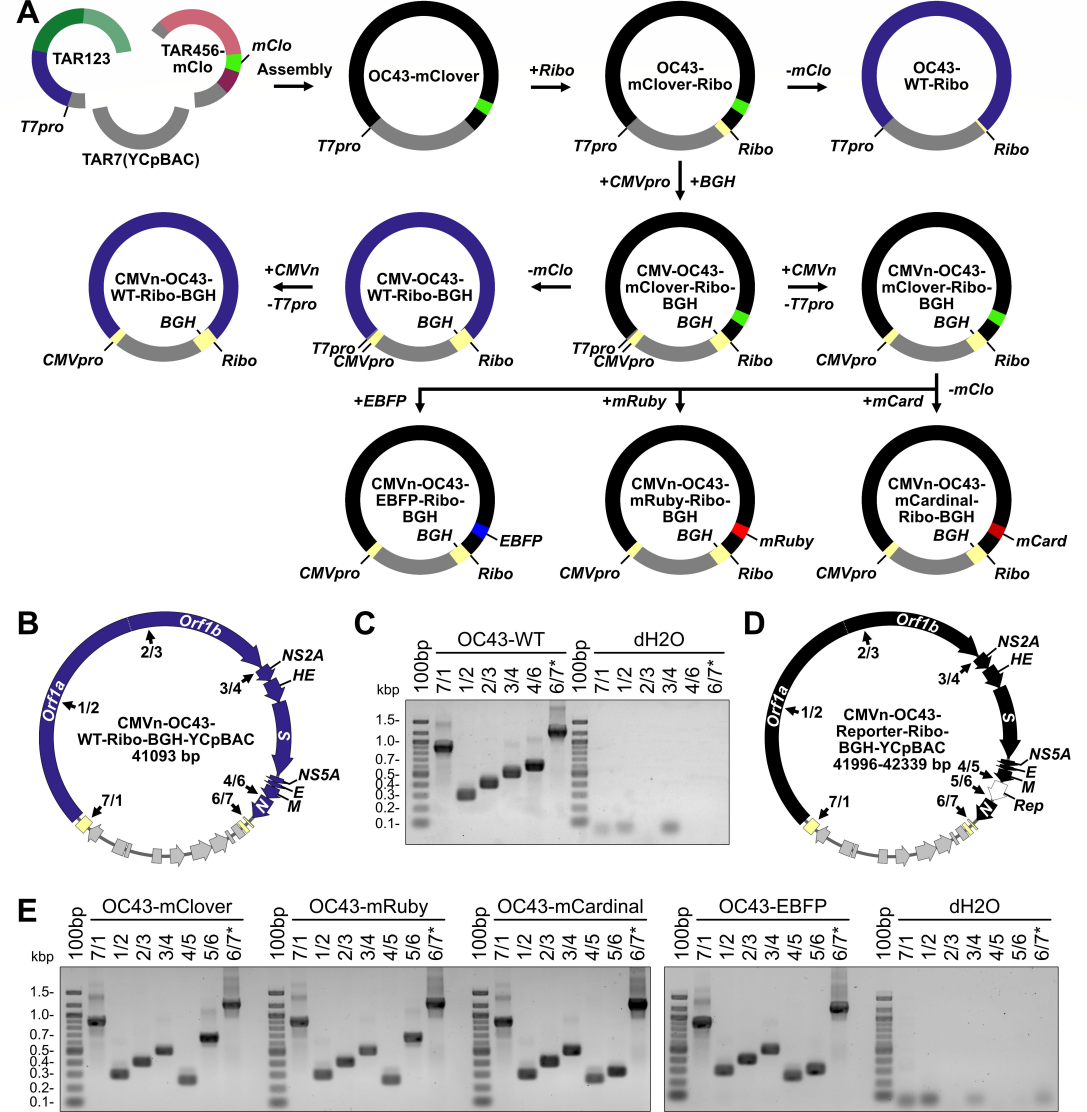
Yeast assembly of full-length HCoV-OC43 wild-type and reporter virus plasmids. (**A**) Assembly of full-length HCoV-OC43-mClover viral sequences (black) was performed by sequential assembly steps in yeast. First, restriction digested OC43TAR123-YCpBAC and OC43TAR456-mClover plasmids were assembled, followed by the sequential insertion of hepatitis delta virus ribozyme (*Ribo*), bovine growth hormone poly(A) signal (*BGH*), and CMV promoter (*CMVpro*) sequences (light yellow). Assembly of full-length HCoV-OC43-WT viral sequences (blue) was performed by removing the *mClover3-H2B* (*mClo*, light green) sequence from a previously assembled plasmid. Additional reporter virus plasmids (black) encoding *enhanced blue fluorescent protein 2* (*EBFP*, blue), *mRuby3-H2B* (*mRuby*, red), or *mCardinal* (*mCard*, dark red) were assembled by recombination which simultaneously removed the *mClover3-H2B* sequence and replaced it with a new reporter sequence. (**B**) Plasmid map for the CMVn-OC43-WT-Ribo-BGH-YCpBAC used for virus rescue. Numbered junctions detected by PCR (black arrows) are indicated. (**C**) PCR screening of assembled DNA junctions 7/1, 1/2, 2/3, 3/4, 4/6, and 6/7 for CMVn-OC43-WT-Ribo-BGH plasmid with water controls (dH2O) to the right of the panel. (**D**) Plasmid map for the CMVn-OC43-Reporter-Ribo-BGH-YCpBAC used for virus rescues. Numbered junctions detected by PCR (black arrows) are indicated. The white arrow in the plasmid map indicates the location of the inserted reporter (*Rep*) genes. (**E**) PCR screening of assembled DNA junctions 7/1, 1/2, 2/3, 3/4, 4/5, 5/6, and 6/7* for CMVn-OC43-Reporter-Ribo-BGH plasmids with water controls (dH2O) to the right of the panel. Primer pairs used for screening are listed in Table S2. Abbreviations: 100bp, 100 bp ladder; bp/kbp, base/kilobase pairs; *T7pro*, T7 promoter sequence; WT, wild type.

To assemble the full-length HCoV-OC43 viral sequences, we co-transformed linearized OC43TAR123-YCpBAC, OC43TAR456-YCpBAC, and amplified YCpBAC sequences into yeast (Fig. 3A). Performing full-length assemblies with three fragments consistently yielded more correct transformants than seven-part assemblies (data not shown). During the assembly of HCMV genomes via TAR, similar three-part, “half-genome” assemblies were also more successful than 17 fragment assemblies (48). Both RNA-based and DNA-based approaches have been used to successfully rescue coronaviruses from plasmids. This includes *in vitro* transcription and capping of a genome length transcript for delivery into cells, *T7pro*-driven transcription with ectopically expressed T7 RNA polymerase in plasmid transfected cells, as well as *CMVpro*-driven transcription in plasmid transfected cells. We initially designed our system to generate full-length mRNA from a *T7pro* with a hepatitis delta virus ribozyme (*Ribo*) inserted following the encoded poly(A) sequence (Fig. 3A, top row). However, these constructs failed to yield infectious viruses from transfected *in vitro* synthesized and capped mRNA or from a plasmid-based, co-transfection with T7 RNA polymerase and vaccina virus D1R capping enzyme plasmids (data not shown). Therefore, we transitioned to a DNA-based rescue system using a CMV promoter (*CMVpro*) upstream of the HCoV-OC43 5′ UTR and a bovine growth hormone polyadenylation signal (*BGH*) downstream of the *Ribo* sequence, which were both inserted into the OC43-mClover-Ribo-YCpBAC using homologous recombination in yeast (Fig. 3A, middle row). Our first *CMVpro*-driven constructs containing *CMVpro* and *T7pro* sequences upstream of the 5′ UTR similarly did not yield infectious virus (data not shown). Ultimately, the constructs that yielded infectious HCoV-OC43 lacked the *T7pro* sequence and utilized a CMV enhancer/promoter sequence positioned 15 bp upstream of the 5′ UTR (*CMVn*) as designed by St-Jean et al. during the generation of an HCoV-OC43 BAC (18). The final full-length assemblies of CMVn-OC43-WT-Ribo-BGH-YCpBAC (wild-type sequence; Fig. 3B and C) and the various fluorescent reporter plasmids (CMVn-OC43-mClover-Ribo-BGH-YCpBAC, CMVn-OC43-mRuby-Ribo-BGH-YCpBAC, CMVn-OC43-mCardinal-Ribo-BGH-YCpBAC, CMVn-OC43-EBFP-Ribo-BGH-YCpBAC; Fig. 3D and E) yielded the correct DNA junction amplicons (Table S2) as shown in Fig. 3C and E. The assembly of the firefly luciferase (*FLuc*)-encoding OC43-YCpBAC plasmid is currently ongoing. The completed WT and reporter virus assemblies were validated with Nanopore sequencing and polymorphisms were identified relative to the most highly conserved HCoV-OC43 reference strain listed in GenBank (Table S3). Of the 23 polymorphisms in protein-coding genes, 12 coded for synonymous amino acids. The vast majority of polymorphisms were present in all full-length assemblies, including a three-codon deletion within *NS5A*. Only 8 of 25 total nucleotide polymorphisms (4 of which are silent mutations) were found in the minority of the YCpBACs, indicating that there was minimal sequence drift in the multiple TAR cloning steps and bacterial propagations between the parental CMVn-OC43-mClover-Ribo-BGH-YCpBAC and the subsequent YCpBACs.

### Rescue of yeast-assembled HCoV-OC43 wild-type and reporter viruses

**Fig 4:**
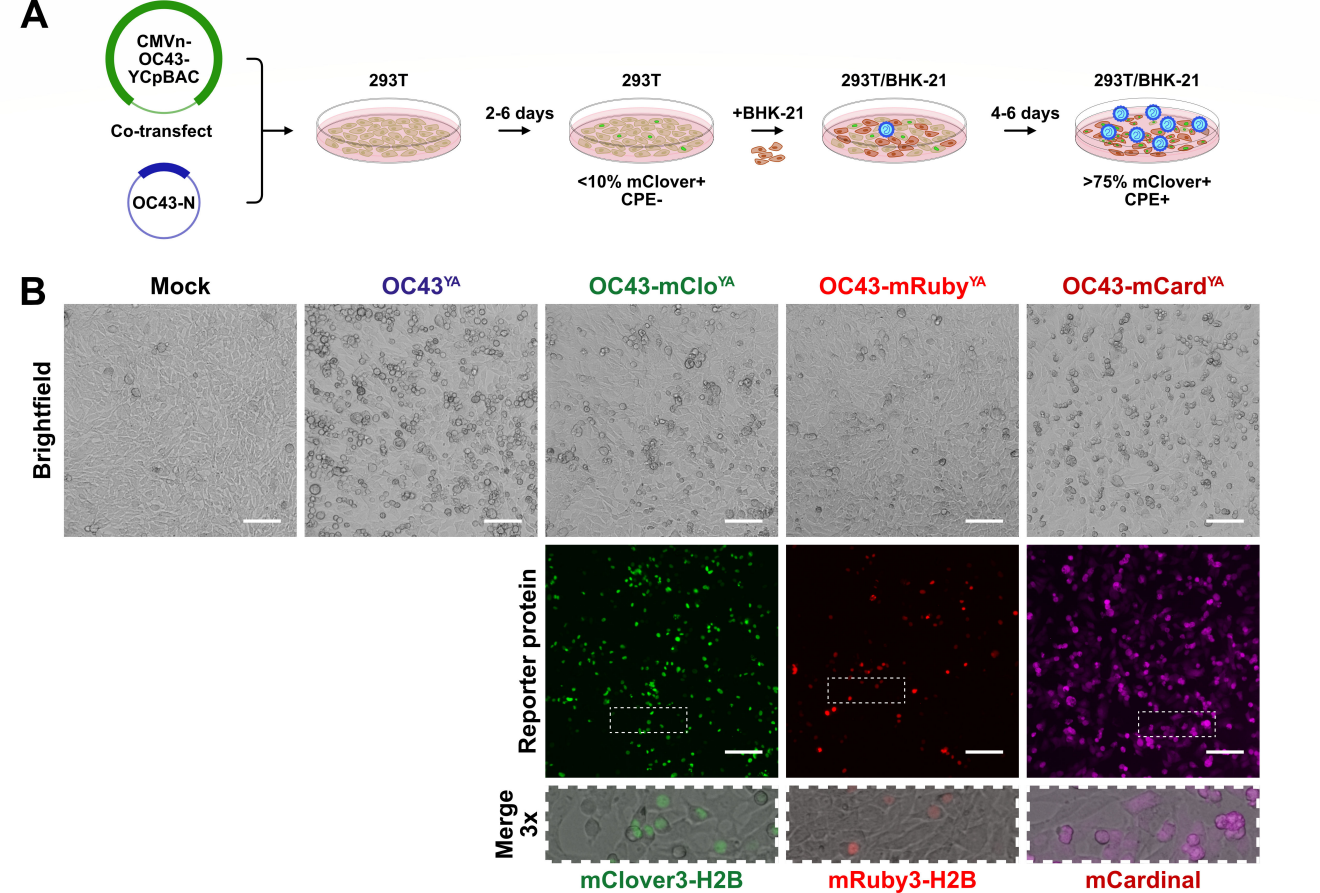
Rescue of OC43^YA^, OC43-mClo^YA^, OC43-mRuby^YA^, and OC43-mCard^YA^ viruses. (**A**) Overview of yeast-assembled OC43 rescue procedure: Assembled CMVn-OC43-YCpBAC plasmids and plasmids encoding OC43-N were co-transfected into 293T (tan) cells for 2–6 days followed by re-seeding with BHK-21 (brown) cells for 4–6 days leading to enhanced cytopathic effect (CPE), reporter protein expression (example showing mClover3-H2B-positive nuclei, green), and virus (blue) propagation. (**B**) Rescue of yeast-assembled viruses: After the 293T/BHK-21 co-culture, cleared culture supernatants were used to infect naïve BHK-21 cells for 24 h prior to fixation and imaging to assess CPE and reporter protein expression in the infected cell monolayers. The areas indicated with the white dashed rectangles are shown at higher magnification to show sub-cellular localization of reporter proteins. Scale bars = 100 µm.

To recover infectious virus from these constructs, purified OC43-mClover, OC43-mRuby, OC43-mCardinal, or OC43-WT YCpBACs were co-transfected into 293T cells with a plasmid encoding *OC43-N* (Fig. 4A), as co-expression of N protein during the initial stages of coronavirus rescue supports virus rescue (15–17, 61). The transfected 293T cells were then co-cultured with more highly permissive BHK-21 cells (62, 63) to promote amplification of the infectious virus (Fig. 4A). The co-culture supernatants were then used to infect naïve BHK-21 cells, which showed clear cytopathic effects (CPE) from infection with all yeast-assembled viruses by 24 hpi (Fig. 4B). Infection with reporter viruses yielded mClover3-H2B or mRuby3-H2B-positive nuclei or diffuse mCardinal expression in the infected monolayers (Fig. 4B). Rescue of a yeast-assembled, HCoV-OC43 expressing EBFP is currently ongoing. The CMVn-OC43-EBFP-Ribo-BGH-YCpBAC plasmid did contain the most unique polymorphisms, single missense mutations in *Orf1a* and *S,* and a polymorphism in the 3′ UTR (Table S3), which could explain our inability to rescue the OC43-EBFP^YA^ virus from this plasmid.

### OC43-mClo^YA^ can replicate to similar titers to wild-type virus in 293A and MRC-5 cells but not HCT-8 cells

**Fig 5:**
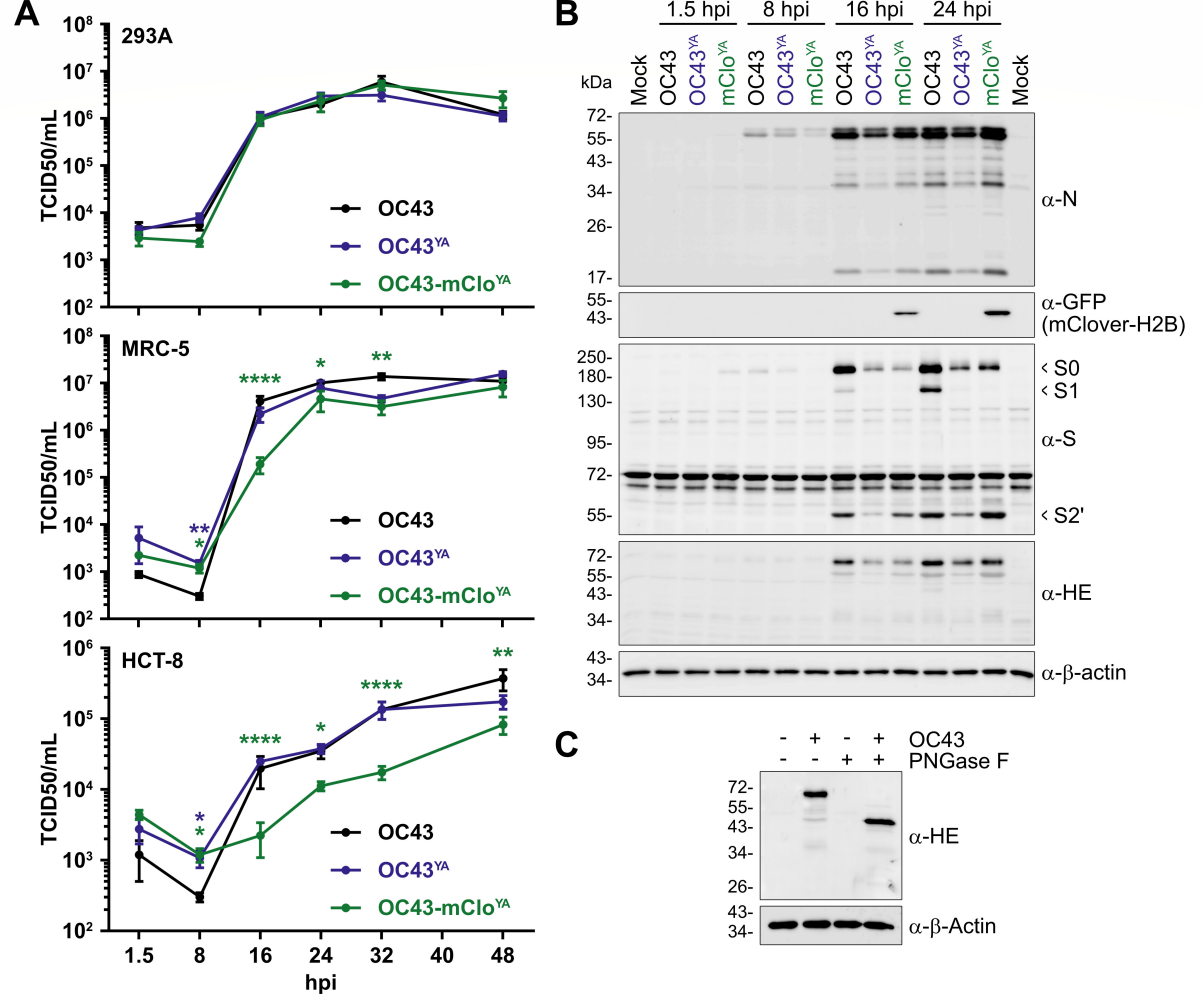
Yeast-assembled HCoV-OC43 viruses replicate to comparable titers as wild-type HCoV-OC43 viruses and produce similar amounts of viral proteins. (**A**) Third passage OC43^YA^ and OC43-mClo^YA^ viruses were used to infect 293A cells (top), MRC-5 cells (middle), or HCT-8 (bottom) at a multiplicity of infection (MOI) for 0.1 and the supernatants were collected at the indicated times and titered using BHK-21 cells. The data are plotted as the mean ± standard error of the mean from four independent replicates. Statistically significant differences compared to OC43(ATCC) are indicated from two-way ANOVA analysis. (**B**) 293A cells were infected at an MOI of 0.1 with OC43 (black), OC43^YA^ (yeast-assembled OC43; blue), or OC43-mClo^YA^ (yeast-assembled OC43-mClover; green) viruses for the indicated times. Protein lysates were subjected to SDS-PAGE and immunoblotted with the indicated antibodies where α-GFP antibodies were used to detect mClover3-H2B. The data presented are from one of four independent experiments. (**C**) Lysates from 293A cells infected with OC43 (24 hpi) were incubated with or without PNGase F prior to immunoblotting with antibodies against OC43-HE or β-Actin. Abbreviations: *P ≤ 0.05; **P ≤ 0.01; ****P ≤ 0.0001; GFP, green fluorescent protein; hpi, hours post-infection; PNGase F, peptide:N-glycosidase F; S0/S1/S2′, Spike subunit 0/1/2′; TCID50, 50% tissue culture infectious dose.

Compared to the ATCC strain of HCoV-OC43 (OC43), OC43-mClo^YA^ and OC43^YA^ replicated with similar kinetics over a 48 h time course of infection in 293A cells (Fig. 5A, top), where no statistically significant differences in titer were observed for either yeast-assembled virus compared to the reference strain of OC43. When replication kinetics were assessed in normal lung fibroblasts (MRC-5 cells), OC43-mClo^YA^ exhibited a moderate replication defect during the early stages of infection compared to OC43(ATCC) infected cells (Fig. 5A, green asterisks). While these delayed replication kinetics persisted through the late stages of infection, ultimately by 48 hpi the titers produced by OC43-mClo^YA^ infected cells equaled those from OC43(ATCC)-infected cells (Fig. 5A, middle). Only at the eight hpi time-point was there a statistically significant difference in OC43^YA^ titer when compared to OC43(ATCC) (Fig. 5A, blue asterisks). Overall, both wild-type viruses replicated similarly in both cell lines, with the titers produced from MRC-5 cells plateauing earlier than those from 293A cells. All viruses did not replicate as robustly in HCT-8 cells as compared to 293A and MRC-5 cells, with titers at 48 hpi being roughly 1.5 log_10_ lower by 48 hpi (Fig. 5A). The OC43-mClo^YA^ virus experienced a sustained replication defect throughout the 48 h time course in HCT-8 cells (Fig. 5A). It is possible that OC43-mClo^YA^ infection in HCT-8 cells may require additional time past 48 h to produce titers comparable to wild-type viruses.

We next more thoroughly assessed the kinetics of viral RNA and protein production during infection with our yeast-assembled viruses in 293A cells. We assessed viral protein production over the first 24 h of infection in 293A cells using HCoV-OC43-specific antibodies to N, S, and HE and a GFP antibody to detect mClover3-H2B expression (Fig. 5B). N protein accumulation (full length,~55 kDa) was observed as early as 8 hours post-infection (hpi) for all viruses and continued to increase over time with minimal differences in expression levels. This demonstrated that TRS* in the OC43-mClo^YA^ virus (Fig. 2D) did not significantly affect N protein production. In addition, numerous smaller proteoforms of N (< 50 kDa) are produced from the yeast-assembled viruses and similar to those observed during OC43(ATCC) infection, which is consistent with previous observations from infections with various coronaviruses (64–67). The OC43(ATCC) virus produced noticeably more HE and S than either of the two yeast-assembled viruses. Multiple S isoforms were detected using the polyclonal S antibody, which are predicted to represent the S0, S1, and S2′ species based on their apparent molecular masses and likely arise from host protease processing of the S protein (Fig. 5B and [68]). We confirmed that the rabbit polyclonal HE antibody was specific for HE, as the protein was only detectable in infected cells and was sensitive to PNGase F treatment (Fig. 5C), consistent with previously reported *N*-glycosylation of HE (69, 70). We noted that despite being governed by identical TRS-B elements (compare TRS and TRS*, Fig. 2D), mClover3-H2B proteins were only detectable at 16 hpi by western blot, whereas N protein accumulated much earlier (Fig. 5B). Despite the observed differences in viral protein accumulation during viral replication, ultimately there was minimal impact on the amount of infectious virus released from 293A cells (Fig. 5A).

### Insertion of an eighth sgRNA sequence alters coronavirus RNA transcriptional efficiencies

**Fig 6:**
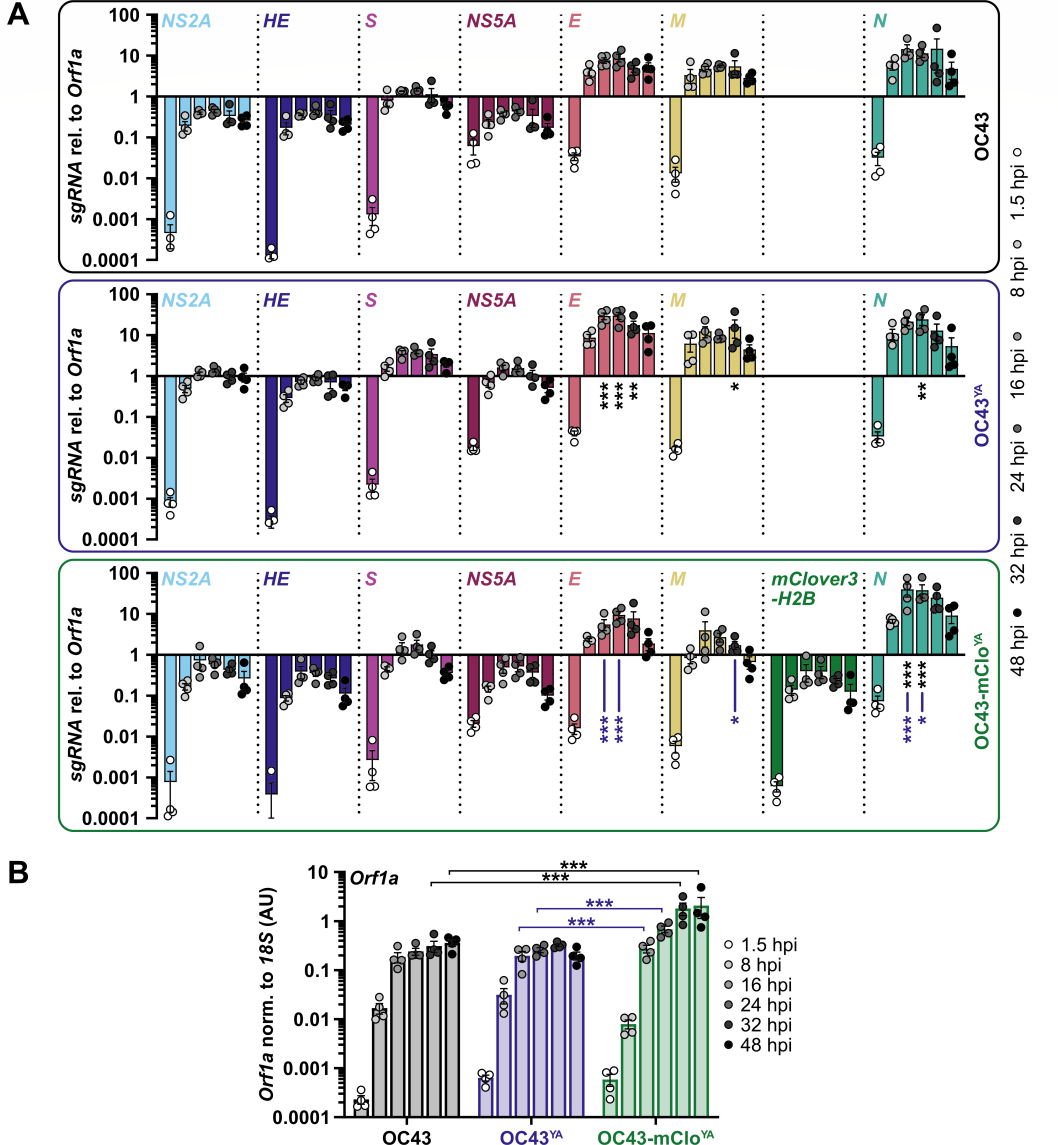
gRNA/sgRNA accumulation is altered by the insertion of the *mClover3-H2B* reporter gene. (**A**) 293A cells were infected at a multiplicity of infection (MOI) of 0.1 with OC43 (black box/top panel), OC43^YA^ (yeast-assembled OC43; blue box/middle panel), or OC43-mClo^YA^ (yeast-assembled OC43-mClover; green box/bottom panel) viruses for the indicated times. Total RNA was reverse transcribed and used for qPCR with a common forward primer that binds in the 5′ leader sequence and reverse primers that bind downstream of the leader (*Orf1a*) or downstream of ORF-specific transcription regulatory sequences. All data were normalized to *18S* rRNA and plotted relative to *Orf1a*. Data were plotted as the mean ± standard error of four independent experiments. Data points < 0.0001 are not shown. (**B**) *Orf1a* (gRNA) abundance normalized to *18S* rRNA expressed as arbitrary units (AU) was plotted as the mean ± standard error of four independent experiments. The same data set was used for panels A and B. Statistical comparisons were made between matched time points by two-way ANOVA analysis. *P* values: *≤ 0.05, **≤ 0.01, ***≤ 0.001. Black asterisks represent differences compared to OC43 and blue asterisks represent differences between OC43^YA^ and OC43-mClo^YA^.

Next, we assessed viral genomic RNA (gRNA) and subgenomic RNA (sgRNA) production during infection with OC43^YA^ and OC43-mClo^YA^ over a 48 h time course in 293A cells (Fig. 6). Reverse transcription was performed using random hexamers and oligo(dT) to amplify all sgRNA species [(-)sgRNA and sg-mRNA]. The qPCRs used a common forward primer for all reactions that anneals in the 5′ leader sequence and unique reverse primers for each viral RNA with the resulting data normalized to *18S* rRNA and then plotted relative to *Orf1a* (gRNA). Overall, the trends in viral RNA production in cells infected with the three viruses were similar: The sgRNA transcripts proximal to *Orf1ab* (*NS2A*, *HE*, *S*, and *NS5A*) were at or below gRNA levels, whereas the sgRNAs encoding *E*, *M*, and *N* were higher in abundance than gRNA (Fig. 6A). This is consistent with previously published RT-qPCR and transcriptomic analysis of HCoV-OC43 infection (71, 72). There were some differences in sgRNA accumulation at specific time points for both the OC43^YA^ and OC43-mClo^YA^ viruses compared to sgRNA accumulation from the OC43(ATCC) virus (Fig. 6A, indicated asterisks). The yeast-assembled viruses produced higher levels of *E* (OC43^YA^), *M* (OC43^YA^), and *N* (OC43^YA^ and OC43-mClo^YA^) transcripts between 16 and 32 hpi when compared to the OC43(ATCC) infections (Fig. 6A, black asterisks). When comparing the two yeast-assembled viruses, the OC43-mClo^YA^ virus produced less *E* and more *N* sgRNAs between 16 and 24 hpi and less *M* sgRNAs at 32 hpi (Fig. 6A, blue asterisks). In addition, gRNA production (*Orf1a*, normalized to *18S* rRNA levels) was ~6-fold higher in OC43-mClo^YA^-infected cells compared to those infected with OC43(ATCC) at 32 and 48 hpi (Fig. 6B, black asterisks) and slightly elevated in OC43-mClo^YA^-infected cells compared to OC43^YA^ gRNA levels in infected cells at 16 and 24 hpi (Fig. 6B, blue asterisks). The insertion of the reporter gene sequence and TRS* into the OC43-mClo^YA^ genome only moderately affected viral transcription (Fig. 6A). The *mClover3-H2B* sgRNA, which is produced from the authentic *N* TRS, only accumulated to levels equivalent to the *Orf1ab*-proximal genes *NS2A*, *HE*, *S*, and *NS5A*. This was unexpected considering the TRS sequence used (Fig. 2D) and the location of the *mClover3-H2B* insertion near the 3′ end of the genome which should allow for high sgRNA transcription (73, 74).

### OC43-mClo^YA^ allows for efficient and sensitive monitoring of viral replication

**Fig 7:**
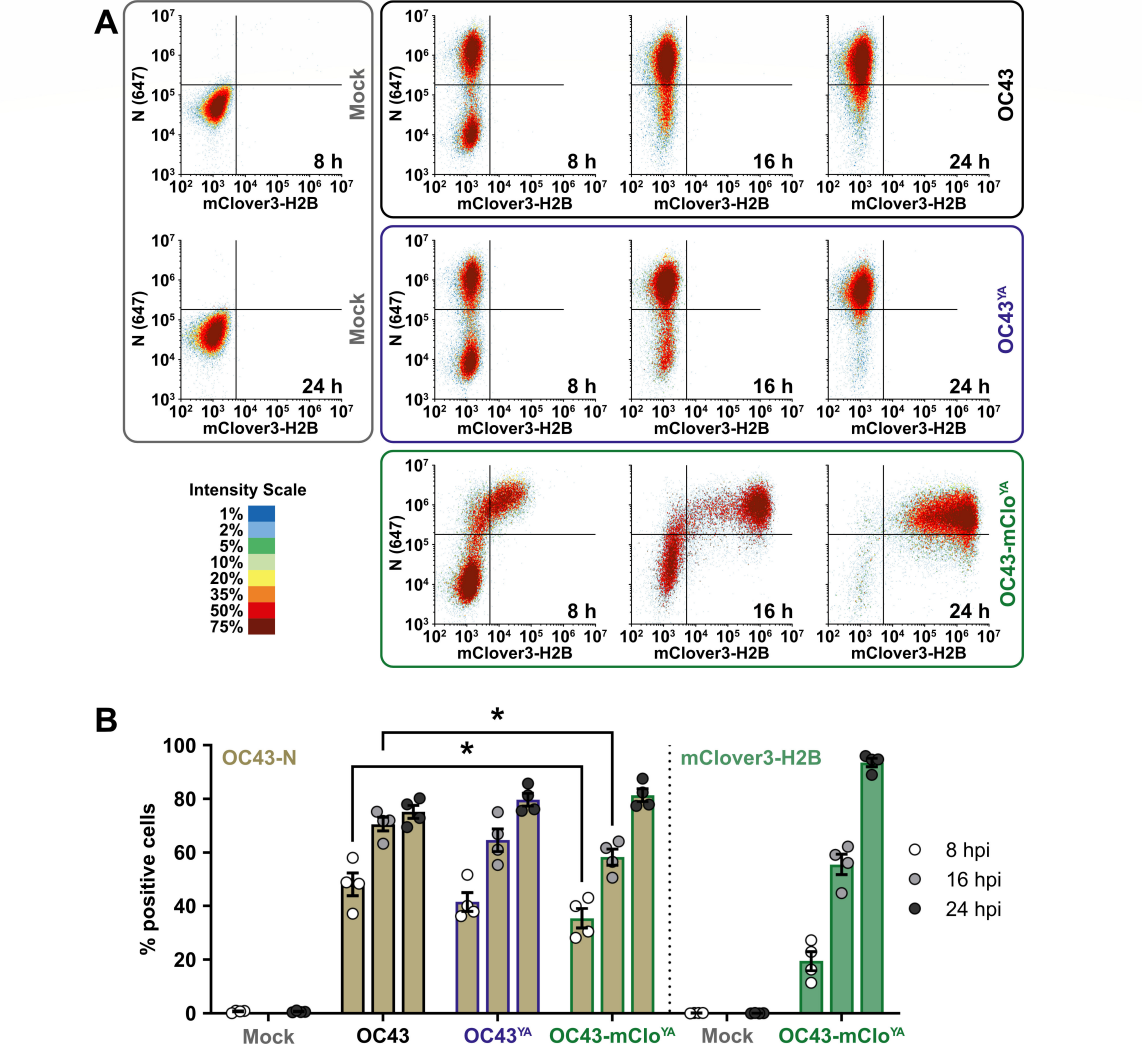
N protein accumulation is delayed during OC43-mClo^YA^ infection. (**A**) 293A cells were infected at an MOI of 0.1 with OC43 (black box/top panel), OC43^YA^ (yeast-assembled OC43; blue box/middle panel), or OC43-mClo^YA^ (yeast-assembled OC43-mClover; green box/bottom panel) viruses for 8, 16, or 24 hours post-infection (hpi) or mock infected for 8 or 24 hours (gray box/left panel). Cells were fixed, permeabilized, and stained with OC43-N antibodies prior to analysis by flow cytometry. Density plots of N (y-axis) vs. mClover3-H2B (x-axis) are shown from one representative experiment. (**B**) The data were analyzed for % positive single cells expressing OC43-N (light brown) or mClover3-H2B (light green) and plotted as the mean ± standard error of the mean from four independent experiments. Statistically significant differences in protein expression between different viruses are indicated (two-way ANOVA), *P ≤ 0.05. Abbreviations: 647, Alexa Fluor 647.

To more quantitatively measure viral replication kinetics, we used flow cytometry to measure N and mClover3-H2B protein expression in infected cells (Fig. 7). Cells from OC43(ATCC), OC43^YA^, or OC43-mClo^YA^ infections (multiplicity of infection [MOI] 0.1) were harvested at the same time points used for western blot analysis (Fig. 5B). These cells were fixed and stained with an antibody against OC43-N and fluorescence thresholds (gates) were set to yield <1% Alexa Fluor 647- or mClover3-H2B-positive mock-infected cells. In all three infections, N expression was easily detectable by 8 hpi and began to plateau by 24 hpi (Fig. 7). The OC43-mClo^YA^ virus produced less N protein at 8 hpi and 16 hpi time points compared to the OC43(ATCC) virus but showed no N expression defect by 24 hpi. This flow cytometry approach was more sensitive compared to western blotting as we observed mClover3-H2B accumulation as early as 8 hpi, after which the fluorescence steadily increased over time. This allowed us to measure the accumulation of an N/mClover3-H2B double-positive population. In line with the RT-qPCR analysis (Fig. 6A), flow cytometry demonstrated that the mClover3-H2B protein has delayed expression kinetics relative to N protein and that the OC43-mClo^YA^ virus exhibited delayed accumulation of N protein relative to the OC43(ATCC) virus (Fig. 7B). However, by 24 hpi, N protein expression from the OC43-mClo^YA^ virus was equivalent to that of OC43(ATCC) and OC43^YA^ and endogenous mClover3-H2B expression more reliably identified infected cells (94% mClover+) compared to exogenous antibody staining (76% N+). This demonstrates that flow cytometric analysis of HCoV-OC43 infection is more sensitive than analysis by western blotting (compare Fig. 5B and 7B) and the OC43-mClo^YA^ reporter virus provides a more streamlined strategy for tracking infectivity in live cells or with only a fixation step needed prior to routine analysis.

**Fig 8:**
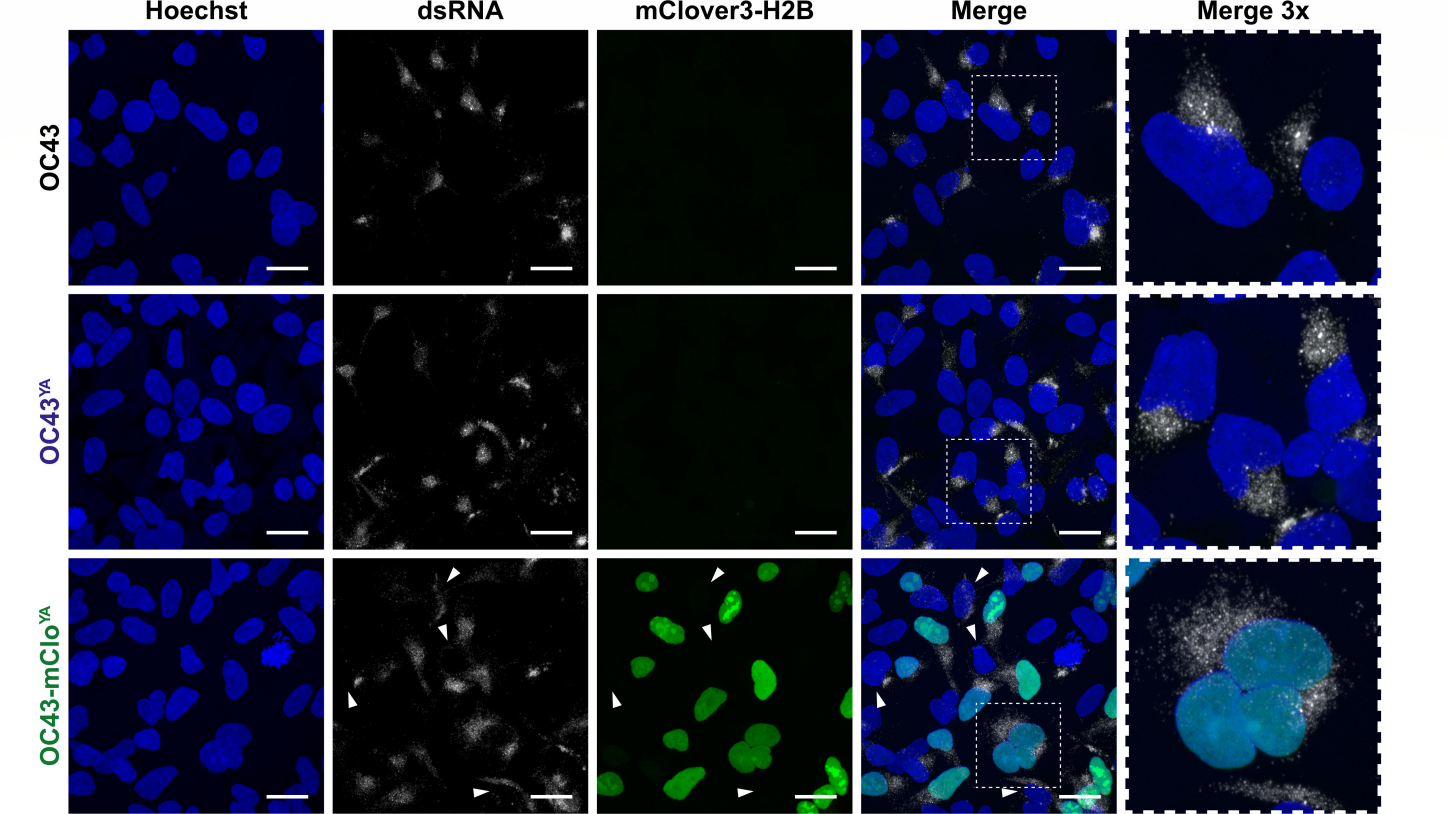
Replication compartment formation is unimpaired in cells infected with yeast-assembled HCoV-OC43 viruses. 293A cells were infected at an MOI of 0.1 with OC43, OC43^YA^ (yeast-assembled OC43), or OC43-mClo^YA^ (yeast-assembled OC43-mClover) viruses for 16 hours prior to fixation, permeabilization, and staining with anti-dsRNA antibodies to stain replication compartments (white cytoplasmic puncta) and Hoechst 33342 to stain cell nuclei (blue). mClover3-H2B is shown in green. White arrowheads denote dsRNA+/mClover- cells. The areas indicated with the white dashed rectangles are shown at higher magnification to show cytoplasmic dsRNA staining. All images were acquired using a confocal laser scanning microscope and are presented as maximum intensity projections from one of three independent experiments. Scale bars = 20 µm. Abbreviations: dsRNA, double-stranded RNA; H2B, histone H2B.

The reporter gene in the OC43-mClo^YA^ virus encodes a mClover variant which was engineered for enhanced photostability and brightness compared to EGFP (53). In addition to these beneficial properties, we also chose the mClover3-H2B fusion protein because it localizes exclusively to nuclei following expression (53). This is particularly advantageous when studying coronavirus biology by microscopy as the mClover3-H2B protein will not interfere with visualizing processes related to the cytoplasmic replication of coronaviruses. We used immunofluorescence microscopy to evaluate cytoplasmic replication compartment formation in cells infected with our WT strains or the OC43-mClo^YA^ virus. Using cells infected with an MOI of 0.1 for 16 h, we observed clear formation of replication compartments for all viruses based on dsRNA staining (Fig. 8). In cells infected with our OC43-mClo^YA^ virus, there were instances of dsRNA staining in cells that did not have detectable mClover3-H2B expression (Fig. 8, white arrowheads). This could be a consequence of the delayed mClover3-H2B expression kinetics compared to replication compartment formation. Future live cell imaging experiments are needed to determine if all dsRNA-positive cells eventually acquire mClover fluorescence.

### Rapid evaluation of antiviral efficacy using OC43-mClo^YA^

**Fig 9:**
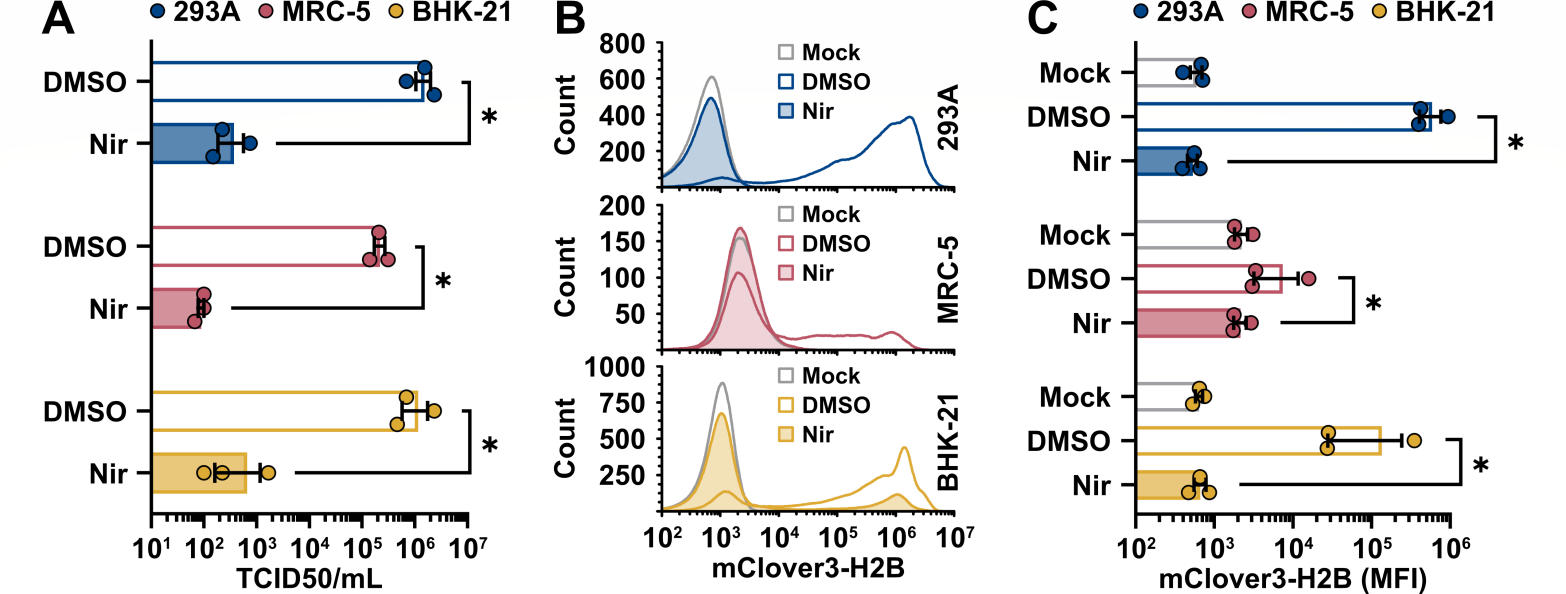
OC43-mClo^YA^ is susceptible to antiviral treatment. 293A, MRC-5, or BHK-21 cells were mock infected or infected at an MOI of 0.1 with OC43-mClo^YA^ (yeast-assembled OC43-mClover) for 1 hour prior to a medium change to 2.5% DMEM+ Pen/Strep/Gln supplemented with 0.1% DMSO or 1 µM nirmatrelvir (Nir). At 24 hours post-infection, the supernatants were collected for titering on BHK-21 cells (**A**). The cell monolayers were collected and fixed prior to analysis of mClover3-H2B expression by flow cytometry (**B**) with the average mClover3-H2B median fluorescence intensity (MFI) from mock or infected cells plotted in panel (C). Data were plotted as the mean ± standard error of three independent experiments. Statistical comparisons were made by two-way ANOVA analysis. *P* values: * ≤ 0.05. Abbreviations: DMSO, dimethyl sulfoxide; TCID50, 50% tissue culture infectious dose.

Reporter viruses are useful tools for antiviral drug discovery. We developed a unique OC43-mClo^YA^ reporter virus, in part, to accelerate low-cost screening of novel compounds in a medium to high-throughput manner. To evaluate the OC43-mClo^YA^ virus as a screening tool for antiviral efficacy, we infected 293A, MRC-5, and BHK-21 cells at an MOI of 0.1 and treated these cells at 1 hpi with nirmatrelvir (PF-07321332), a potent coronavirus antiviral that targets Mpro (75). Matched cells and culture supernatants were used to monitor viral replication by flow cytometry and titering on BHK-21 cells, respectively. The OC43-mClo^YA^ virus infected and replicated in all cell lines tested (Fig. 9). Moreover, nirmatrelvir reduced viral titers by ~3 log_10_ in all cell lines tested (Fig. 9A). These effects were mirrored by the flow cytometric analysis that showed clear reductions in the fluorescence intensity of infected cells following nirmatrelvir treatment (Fig. 9B and C). These data are consistent with the known antiviral activity of nirmatrelvir against HCoV-OC43 (75–77). The OC43-mClo^YA^ virus did not infect MRC-5 cells as readily as 293A or BHK-21 cells based on the intensity of mClover3-H2B staining in these cell populations (Fig. 9B and C), which limited the dynamic range for antiviral testing in MRC-5 cells with this reporter virus. Infection of the 293A and BHK-21 cells with OC43-mClo^YA^ produced far more and far brighter mClover +cells compared to MRC-5 cells (Fig. 9B and C). Despite these differences in reporter expression, OC43-mClo^YA^ still replicated to high titers in the MRC-5 cells (Fig. 9A). These data together provide a proof-of-principle for the use of the OC43-mClo^YA^ virus for streamlined antiviral testing and highlight the utility of synthetic biology techniques for the generation and study of mutant coronaviruses.

## DISCUSSION

Once established, reverse genetics systems provide well-defined clonal genetic material that can be readily propagated and mutagenized for fundamental studies of viral biology and a variety of biomedical applications. Here, we report the creation of an updated reverse genetics system for the endemic, seasonal, betacoronavirus HCoV-OC43. We used TAR methodology to capture fragments of the ~31 Kb (+)ssRNA HCoV-OC43 genome to store as sequence-validated dsDNA parts. Combinatorial assembly of the dsDNA parts in yeast yielded an intact dsDNA copy of the HCoV-OC43 genome that could initiate a full viral gene expression program upon introduction into cell lines (Fig. 4). Moreover, we provide proof-of-concept for the engineering of HCoV-OC43 reporter viruses encoding additional sgRNAs without altering the coding regions of neighboring coronavirus genes. We thoroughly evaluated the yeast-assembled, recombinant HCoV-OC43 viruses, demonstrating comparable viral gene expression, susceptibility to antivirals, and fitness in cell culture compared to its natural progenitor (Fig. 4 to 9). We did observe a cell type-dependent inhibition in the replication of our OC43-mClo^YA^ reporter virus relative to wild-type viruses (Fig, 5A). It will be interesting to determine with future experiments if these phenotypes are dependent on the specific innate immune responses inherent to each cell type and if so, if the dysregulation in viral RNA expression from our reporter virus is responsible. Our future experiments will determine if these replication delays are shared with our other reporter viruses (OC43-mRuby^YA^ and OC43-mCard^YA^) or if there is a reporter sequence- or position-dependent component to the phenotype. Our approach to coronavirus genome assembly provides a viable path to generate recombinant HCoV-OC43 viruses.

Coronaviruses rely on discontinuous transcription to create (-)sgRNAs that are converted into sg-mRNAs that encode structural proteins and accessory proteins. This process relies on sequence complementarity between nascent (-)sgRNA TRS-B hairpin sequences and parental (+)gRNA TRS-L hairpin sequences, which enables template switching by the viral RdRp. Despite evidence that the RdRp favors the generation of short (-)sgRNA by template switching at the first TRS-B that it encounters, the precise rules that govern template-switching preferences and the ultimate stoichiometry of sgRNA products remain poorly understood. Considering the similarity of TRS-B sites across the HCoV-OC43 genome, we reasoned that insertion of a reporter gene linked to its own TRS-B should enable efficient production of an additional sg-mRNA encoding the reporter gene (Fig. 2D). Our study demonstrated the feasibility of this approach for HCoV-OC43, whereby linking the reporter gene in the OC43-mClo^YA^ virus to the *N* TRS-B resulted in efficient mClover3-H2B expression (Fig. 7), with minimal effects on N expression (Fig. 5B and 7B) from the *N* sg-mRNA regulated by the 23 base TRS* (Fig. 2D). The OC43-mClo^YA^ virus displayed equivalent replication kinetics and overall fitness in cell culture compared to the OC43(ATCC) virus in 293A cells, with only moderately delayed replication kinetics in MRC-5 cells (Fig. 5A). Our observations were consistent to those made using the MHV system where the TRS/luciferase gene insertions were well-tolerated and recombinant MHVs replicated similarly to wild-type virus with modest attenuation of upstream sgRNA expression (10). This prior work also demonstrated that the genomic position and sequence of the inserted gene can influence reporter expression and stability (10), making it necessary to empirically evaluate future heterologous gene insertions into the HCoV-OC43 genome.

The sgRNAs encoding *mClover3-H2B* accumulated to lower levels than the sgRNAs encoding *E*, *M* and *N* despite being transcribed from an adjacent TRS-B (Fig. 6). We speculate that this difference in sgRNA levels could stem from less efficient template switching at the *mClover3-H2B* TRS-B compared to the other authentic TRS-Bs, perhaps because of RNA sequences between the TRS and *mClover3-H2B* start codon that resulted from our reporter cloning strategy. Interestingly, this conclusion differs from those of previous studies using mouse hepatitis virus defective interfering (DI) RNA constructs that demonstrate that inserted sequences of up to ~1.5 kb upstream or downstream of a DI RNA TRS had little impact on sgRNA production (78). This suggests there are differences in how the RdRp interacts with TRS-flanking sequences in the context of the full-length gRNA compared to artificial DI RNA constructs. Coronavirus sgRNA abundance is inversely proportional to the length and the number of internal TRSs present within the transcript (73, 74). We did not observe a stepwise reduction in upstream sgRNA/gRNA abundance during infection with the OC43-mClo^YA^ virus that one might expect from the insertion of an additional TRS-B; conversely gRNA levels were increased in OC43-mClo^YA^-infected cells compared to WT virus infection (Fig. 6B). The unexpected regulation of *mClover3-H2B* sgRNA transcripts would suggest that there are more complex regulatory processes at play during authentic viral infection. Future work will alter the RNA sequence present in the OC43-mClo^YA^ genome to attempt to enhance reporter gene expression while also providing insight into how TRS context affects coronavirus transcription.

A benefit of the TAR methodology is the flexibility of altering viral sequences through fragment-mediated assembly. This allows for the rapid generation of recombinant viruses from clinical isolates as demonstrated with SARS-CoV-2 and HCMV (45, 48). Not only could this allow for the study of emerging viruses with reverse genetics, but it may also allow for the swapping of sequences to study gene variants or strain-specific differences; similar to the methodology employed in this paper to exchange reporter genes (Fig. 3A). HCoV-OC43, as well as other human coronaviruses, circulate in a recurrent, seasonal pattern due to short-lived host immunity (79, 80). This can be attributed, in part, to adaptive evolutionary changes observed in the HCoV-OC43 Spike protein sequence over time (81), where the presence of HCoV-OC43 antibodies can reduce the severity of subsequent coronavirus infection (82, 83). The TAR methodology allows for the generation of pools of HCoV-OC43 viruses based on naturally occurring strains to determine which strains elicit superior cross-neutralizing antibody responses in animal models for possible vaccine development. Likewise, naturally occurring sequences could be engineered into HCoV-OC43 genomes via TAR to test for resistance to current or novel direct-acting antivirals.

## MATERIALS AND METHODS

### Mammalian cells and viruses

Human embryonic kidney 293A, human embryonic kidney 293T, and human lung fibroblast MRC-5 cells were grown in Dulbecco’s modified Eagle’s medium (DMEM; Thermo Fisher, 11965118) supplemented with heat-inactivated 10% fetal bovine serum (FBS, Thermo Fisher, A31607-01), 100 U/mL penicillin, 100 µg/mL streptomycin, and 2 mM L-glutamine (Pen/Strep/Gln; Thermo Fisher, 15140122 and 25030081). Human epithelial adenocarcinoma HCT-8 cells were maintained as above yet supplemented with 1× MEM non-essential amino acids (Thermo Fisher, 11140050). Baby hamster kidney (BHK-21) cells were maintained in DMEM supplemented with 5% FBS and Pen/Strep/Gln. All cells were maintained at 37°C in a 5% CO_2_ atmosphere.

Stocks of human coronavirus OC43 (HCoV-OC43; ATCC, VR-1558) and recombinant HCoV-OC43 were propagated in BHK-21 cells. Cells were infected at a MOI of 0.05 for 1 h at 37°C in serum-free DMEM. After 1 h, the infected cells were maintained in DMEM supplemented with 1% FBS and Pen/Strep/Gln until the cytopathic effect was complete. Upon harvest, the culture supernatant was centrifuged at 1,000 × *g* for 5 min at 4°C, aliquoted, and stored at −80°C. Viral titers were measured using median tissue culture infectious dose (TCID50) assays. Following the five-fold serial dilutions of the samples, BHK-21 cells were infected for 1 h at 37°C, followed by the replacement of inocula with 1% FBS in DMEM + Pen/Strep/Gln. Viral titers were calculated using the Spearman-Kärber method (84).

### Plasmids

The plasmid pCC1-BACYCp-URA3 (YCpBAC; [57]) was used for all transformation-associated recombination (TAR) cloning. The plasmids pKanCMV-mClover3-10aa-H2B (mClover3 fused to a C-terminal histone H2B; Addgene, Plasmid#74257 [53]), pKanCMV-mRuby3-10aa-H2B [mRuby3 fused to a C-terminal histone H2B; Addgene, Plasmid#74258 (53)], mCardinal-C1 (Addgene, Plasmid#54799 [85]), EBFP2-C1 [Addgene, Plasmid#54665 (86)], and FLuc Control Template (NEB) were used as templates for amplification of the respective reporter ORFs. The CMV promoter (CMVpro) and BGH poly(A) signal (BGH) sequences were obtained from pcDNA3.1(+). The TAR cloning plasmids pUC57-N-TRS and pUC57-HDV-T7term encoding the *OC43-N* TRS or hepatitis delta virus (HDV) ribozyme and T7 terminator sequences, respectively, were synthesized by GenScript. A codon-optimized HCoV-OC43 N expression plasmid, pTwist-OC43-N(CO) (Addgene, Plasmid#151960), was co-transfected with assembled HCoV-OC43 plasmids during the viral rescue with 293T cells. All plasmids generated for this study are included in Table S4.

### Yeast and bacterial cell propagation and transformation

*Saccharomyces cerevisiae* strain VL6-48N (*MATα*, *his3-Δ200*, *trp1-Δ1*, *ura3-Δ1*, *lys2*, *ade2-101*, *met14*, *psi +cir^0^*) (87) was used for all yeast transformation experiments following previously established protocols (48, 88) with the following changes: Yeast cells were grown in yeast extract peptone dextrose (YEPD) medium to a density of 2–4 × 10^7^ cells/mL, the working concentration of zymolyase (80 µg/mL; MP Biomedicals, 08320921) was empirically determined to yield near complete spheroplast generation after 30–40 min as determined by visualization of spheroplasts in 1 M sorbitol compared to those in 2% SDS using a hemocytometer, each 200 µL spherolplast suspension contained: <50 ng amplified pCC1BACYCp-URA (YCpBAC) plasmid (linear capture plasmids), <3 µg linear viral genomic cDNA amplicons/restriction fragments and/or recombinant DNA, and 5–10 µg of salmon sperm DNA (Thermo Fisher, 15632011), and incubations in PEG 8000 solution or SOS solution were 20 min and 30 min, respectively. Transformed spheroplasts resuspended in melted SORB-TOP-URA and plated onto SORB-URA plates prior to incubation at either room temperature or 30°C until colonies formed (88).

*Escherichia coli* strain Stbl3 (Thermo Fisher, C737303) was used to maintain and propagate all plasmids in bacteria. An overnight culture of these cells was rendered electrocompetent by growing to log-phase followed by three washes and resuspension in ice-cold 10% glycerol. To transfer YCpBACs into Stbl3 *E. coli*, total yeast DNA obtained from cells following overnight liquid culture in SC-URA medium was purified using the Gentra/Puregene Yeast/Bacteria Kit (QIAGEN, 158567). Yeast DNA was electroporated into Stbl3 cells using a Gene Pulser II electroporation system (Bio-Rad) set to 2.5 kV/25 µF/200 Ω with 0.2 cm electroporation cuvettes (VWR, 89047-208).

### Synthesis and capture of HCoV-OC43 genome fragments

To generate YCpBAC plasmids, the HCoV-OC43 genome was divided into five TAR fragments (1, 2, 3, 4, and 6). Corresponding primer pairs (OC43-TARxF/OC43-TARxR, Table S1) for each TAR fragment were used to first amplify the YCpBAC plasmid to generate linear capture plasmids with 45 bp of sequence homology associated with the 5′ and 3′ ends of each viral genomic fragment using KOD Xtreme Hot Start DNA Polymerase (KODpol; Sigma-Aldrich, 71975-M) following the manufacturer’s instructions for two-step cycling. These primers were designed to incorporate either *Not*I (TAR1 and TAR7) or *I-Sce*I (TAR2-6) restriction sites between the YCpBAC and HCoV-OC43 sequences to facilitate the release of HCoV-OC43 sequences for TAR cloning.

Viral RNA from HCoV-OC43 virions (ZeptoMetrix, 0810024CF; ATCC, VR-1558) was isolated using the RNeasy Plus Mini Kit (QIAGEN, 74136) and reverse transcribed into cDNA fragments using gene-specific primers 1/2R, 2/3R, 3/4R, 5/6R (Table S1) or oligo(dT)_20_ and the SuperScript III First-Strand Synthesis System (Invitrogen, 18080–051) with a modified protocol for long cDNA synthesis (89). cDNAs were amplified using PCR with primers TAR1ampF/1/2R (TAR1), 1/2F/2/3R (TAR2), 2/3F/3/4R (TAR3), 3/4F/5/6R (TAR4), or 4/5F/TAR6ampR (TAR6) (Table S1) to generate dsDNA fragments of the HCoV-OC43 genome using KODpol following the manufacturer’s instructions.

To generate YCpBACs containing single TAR fragments, linear capture plasmids were co-transformed into yeast with cDNA amplicons spanning the desired region of the viral genome to generate the plasmids: OC43TAR1-YCpBAC, OC43TAR2-YCpBAC, OC43TAR3-YCpBAC, OC43TAR4-YCpBAC, and OC43TAR6-YCpBAC.

The TAR5(mClover3-H2B) dsDNA fragment was generated by PCR with KODpol using the OC43-TAR5F-mClover/OC43-TAR5R-mClover primers using the pKanCMV-mClover3-10aa-H2B plasmid as a template. This amplicon was digested with *Hind*III and phosphorylated with T4 polynucleotide kinase (New England Biolabs (NEB), M0201S) and cloned into pcDNA3.1+ digested with *Eco*RV and *Hind*III restriction sites to create pcDNA3.1-TAR5-mClover3-H2B. Since the positioning of the TAR5 reporter gene will be downstream of the authentic *N* transcription regulatory sequence (TRS), a second copy of the *N* TRS (TRS*, Fig. 2D) was inserted downstream of the reporter genes to produce the sgRNAs encoding *N* in reporter viruses. A *Bam*HI/*Afl*II restriction fragment containing the duplicated TRS from pUC57-N-TRS was ligated into *Bam*HI/*Afl*II digested pcDNA3.1-TAR5-mClover3-H2B to generate pcDNA3.1-TAR5-mClover3-H2B-TRS. The remaining TAR5 reporter fragments were generated by *Age*I/*Bam*HI digestion of pKanCMV-mRuby3-H2B, mCardinal-C1, EBFP2-C1, or a PCR amplicon of FLuc Control Template (AgeI-TAR5F-FLuc/BamHI-TAR5R-FLuc, KODpol) and ligated into pcDNA3.1-OC43TAR5-mClover3-H2B-TRS cut with the same enzymes to generate: pcDNA3.1-OC43TAR5-mRuby3-H2B-TRS, pcDNA3.1-OC43TAR5-mCardinal-TRS, pcDNA3.1-OC43TAR5-EBFP2-TRS, and pcDNA3.1-OC43TAR5-FLuc-TRS. The wild-type (WT)/reporter-less TAR fragment was generated by PCR of viral cDNA using KODpol and the primers OC43-TAR5F-WT/OC43-TAR5R-WT.

### Screening of yeast assemblies by colony PCR

Yeast colonies from the SORB-URA transformation plates were patched onto SC-URA plates and incubated overnight at 30°C. Patched yeast were lysed in 20 µL 0.05% SDS at 95°C for 15 min or 0.25 mg/mL zymolyase in 50 mM Tris-Cl (pH 7.5)/25% glycerol at 37°C for 15 min. 1–5 µL of lysate was screened using 25 µL *Taq* DNA Polymerase with ThermoPol Buffer (NEB, M0267L) reactions following the manufacturer’s protocol with 500 nM primers (Table S2). PCRs were analyzed following DNA gel electrophoresis using 1.8% agarose/TAE gels with a Wide Mini-Sub Cell GT apparatus and 26-well combs (Bio-Rad, 1704469 and 1704457).

### Assembly of HCoV-OC43 sequences in yeast

Partial assemblies of OC43TAR123-YCpBAC (containing *Orf1ab*) or OC43TAR456—Ruby3-H2B were assembled in yeast using equimolar ratios of the individual TAR plasmids and five-fold less YCpBAC capture plasmid. The individual TAR fragments were linearized for assembly following restriction digestion or PCR amplification using KODpol. A truncated YCpBAC capture plasmid generated by KODpol PCR with OC43-TAR7F-trunc/OC43-TAR7R-trunc primers was used for the partial assemblies. Partial assemblies of OC43TAR456-WT, mClover, mCardinal, EBFP, or FLuc YCpBACs were performed using a *Bsi*WI-digested OC43TAR456-mRuby-H2B-YCpBAC (Fig. 2B) co-transformed with *I-Sce*I-digested pcDNA3.1-OC43TAR5 reporter plasmids. Full assemblies (Fig. 3A) were performed by co-transforming equimolar ratios of *Sgr*AI/*I-Sce*-I-digested OC43TAR123-YCpBAC and *Srf*I/*Sgr*AI-digested OC43TAR456-mClover-YCpBAC (Fig. 2A and B) with 10-fold less truncated YCpBAC to generate OC43-mClover-YCpBAC.

Yeast colonies were screened for YCpBACs containing the desired DNA junctions using *Taq* PCRs as indicated above. Completed assemblies were verified by restriction analysis, Sanger sequencing (Genewiz), and Oxford Nanopore sequencing (Plasmidsaurus).

### Insertion of transcriptional control and RNA modification elements using TAR cloning

Following assembly of the full-length OC43-mClover-YCpBAC, sequences encoding the HDV ribozyme and T7 terminator were inserted immediately 3′ of the encoded poly(A) sequence (Fig. 3A). The HDV ribozyme/T7 terminator sequences were excised from pUC57-HDV-T7term using *Esp*3I and co-transformed with the OC43-mClover-YCpBAC digested with *I-Sce*I into yeast for TAR assembly to generate OC43-mClover-Ribo-YCpBAC. *Bam*HI/*I-Sce*-I-digested OC43-mClover-YCpBAC was co-transformed with a PCR amplicons containing WT HCoV-OC43 sequence spanning the *M-N* genes (4/5F/5/6R, OC43 cDNA, KODpol) and the sequences encoding the HDV ribozyme/T7 terminator (6/7F/6/7R, OC43-mClover-Ribo-YCpBAC) to generate OC43-WT-Ribo-YCpBAC (Fig. 3A).

To modify the YCpBACs for mammalian cell rescue, CMVpro and BGH sequences were inserted into OC43-mClover-Ribo-YCpBAC (Fig. 3A). CMVpro: A *Xho*I/*Bgl*II fragment from OC43TAR1-YCpBAC containing the OC43 5’UTR was cloned into pcDNA3.1+ digested with the same enzymes. The resulting plasmid was digested with *Srf*I and a blunted (NEB Quick Blunting Kit, E1201S) *Mlu*I/*Sac*I digest of pcDNA3.1+ (containing the CMVpro sequence) was ligated into the backbone to create pcDNA3.1-CMV-OC43-5’UTR. BGH: pUC19 digested with *Nde*I/*Xba*I was ligated with a *Nde*I/*Xba*I insert containing the pcDNA3.1+ MCS to generate pUC19pc. The *Eco*RI/*Hpa*I *cos*-containing fragment from YCpBAC was ligated into pUC19pc and digested with *Eco*RI/*Pvu*II to create pUC19pc-cos. A PCR amplicon of the pcDNA3.1+ BGH (BD206/BD207, containing BGH, KODpol) was phosphorylated and blunt-end ligated into pUC19pc-cos digested with *Not*I and blunted to generate pUC19pc-cos-BGH. The OC43-mClover-Ribo-YCpBAC was digested with *Not*I and co-transformed into yeast with the *Xho*I/*Bgl*II fragment of pcDNA3.1-CMV-OC43-5′ UTR and the *Sfo*I/*Sal*I fragment of pUC19pc-cos-BGH to generate CMV-OC43-mClover-Ribo-BGH-YCpBAC. To remove the T7 promoter sequence in CMV-OC43-mClover-Ribo-BGH-YCpBAC and shorten the spacing between CMVpro and the OC43 5’UTR (18), a pair of oligonucleotides (CMVn-OC43F/CMVn-OC43R) were annealed and filled-in with DNA Polymerase I, Large Fragment (Invitrogen, 18012021) and co-transformed with *Not*I-digested CMV-OC43-mClover-Ribo-BGH-YCpBAC to generate CMVn-OC43-mClover-Ribo-BGH-YCpBAC (Fig. 3A).

The WT version of this plasmid was created by co-transforming *Bam*HI-digested CMV-OC43-mClover-Ribo-BGH-YCpBAC with a PCR amplicon of WT HCoV-OC43 sequence (4/5F/6/7R, OC43-WT-Ribo-YCpBAC, KODpol) into yeast to generate CMV-OC43-WT-Ribo-BGH-YCpBAC (Fig. 3A). This plasmid was then digested with *Not*I and co-transformed with annealed and filled-in CMVn-OC43F/CMVn-OC43R to generate CMVn-OC43-WT-Ribo-BGH-YCpBAC (Fig. 3A).

The CMVn-OC43-mClover-Ribo-BGH-YCpBAC was digested with *Bam*HI was co-transformed into yeast with PCR amplicons containing the *EBFP2* gene (4/5F/sgN, OC43TAR456-EBFP-YCpBAC, KODpol) and the OC43-3′ UTR/ribozyme sequence (BD205/Ribo_Rev, CMVn-OC43-mClover-Ribo-BGH-YCpBAC, KODpol) to generate the CMVn-OC43-EBFP-Ribo-BGH-YCpBAC plasmid. To generate the CMVn-OC43-mRuby-Ribo-BGH-YCpBAC plasmid, *Pme*I/I-*Sce*I-digested CMVn-OC43-WT-Ribo-BGH-YCpBAC was co-transformed into yeast with *Sal*I/*Sgr*AI-digested OC43TAR456-mRuby-YCpBAC. To generate the CMVn-OC43-mCardinal-Ribo-BGH-YCpBAC, *Pme*I/*Kfl*I-digested CMVn-OC43-WT-Ribo-BGH-YCpBAC was co-transformed into yeast with I-*Sce*I/*Sgr*AI-digested OC43TAR456-mCardinal-YCpBAC.

Yeast colonies were screened for YCpBACs containing the desired DNA junctions using *Taq* PCRs as indicated above. Completed assemblies were verified by restriction analysis, Sanger sequencing (Genewiz), and Oxford Nanopore whole plasmid sequencing (Plasmidsaurus). Single-nucleotide polymorphisms in CMVn-OC43-WT-Ribo-BGH-YCpBAC (WT), CMVn-OC43-mClover-Ribo-BGH-YCpBAC (mClover), CMVn-OC43-mRuby-Ribo-BGH-YCpBAC (mRuby), CMVn-OC43-mCardinal-Ribo-BGH-YCpBAC (mCardinal), and CMVn-OC43-mClover-EBFP-BGH-YCpBAC (EBFP) plasmids compared to a reference strain of HCoV-OC43 (GenBank accession ON376724) are listed in Table S3.

### Rescue of infectious yeast-assembled HCoV-OC43 recombinant viruses

293T cells seeded at ~500,000 cells per well into 6-well plates were co-transfected with 1 µg of CMVn-OC43-mClover-Ribo-BGH-YCpBAC or CMVn-OC43-WT-Ribo-BGH-YCpBAC and 200 ng pTwist-OC43-N(CO) plasmids diluted in Opti-MEM I (Thermo Fisher, 31985070) and combined with 3.6 µL PEI MAX (linear polyethylenimine (MW 40,000), 1 mg/mL in water (pH 7.4), 0.22 µm filtered; Polysciences, 24765) diluted in Opti-MEM I. Rescue transfections of OC43-mRuby^YA^ and OC43-mCard^YA^ used 2.5 µg YCpBAC plasmid, 500 ng pTwist-OC43-N(CO), and 9 µL PEI MAX. Transfections were performed in serum-free DMEM for 4 h followed by a change of medium to 10% DMEM +Pen/Strep/Gln and the cells were left at 37°C. After 2–4 days, the transfected 293T cells were trypsinized and seeded into 10 cm dishes with BHK-21 cells and co-cultured for 4–6 days in 2.5% DMEM +Pen/Strep/Gln until CPE was visible. Culture supernatants from these co-cultured cells were cleared by centrifugation (500 × *g* for 5 min) and used to infect monolayers of BHK-21 cells for up to 6 days depending on when complete CPE was visible (1st passage). Culture supernatants from these 1st passage infections were cleared by centrifugation and used to infect monolayers of BHK-21 cells for 2 days (2nd passage). These 2nd passage virus stocks were used to infect flasks of BHK-21 cells to generate virus stocks (3rd passage) for subsequent experiments.

### Infections for time course experiments

293A cells were infected with OC43(ATCC), OC43^YA^, or OC43-mClo^YA^ at an MOI of 0.1 in serum-free DMEM for 1 h at 37°C. The inocula were removed at 1 h post-infection (hpi) and replaced with 2.5% DMEM +Pen/Strep/Gln prior to harvest at 1.5, 8, 16, 24, 32, or 48 hpi.

#### Titering

Viral supernatants from infected cells after the media change were collected and stored at −80°C prior to analysis by TCID50 assays as indicated above.

#### RNA samples for RT-qPCR

All medium was removed from the cells and the cells were lysed with 350 µL Buffer RLT Plus (QIAGEN). Cell lysates were stored at −80°C until processed for RNA isolations.

#### Cell lysates for western blotting

All medium was removed from the cells and the cells were gently washed with phosphate-buffered saline (PBS; Thermo Fisher, 10010023). After the PBS was removed, the cells were lysed with 2× Laemmli Buffer (12.5 mM Tris-HCl (pH 6.8), 4% SDS, 20% glycerol), and lysates were passed through QIAshredder columns (QIAGEN, 79654) for 2 min at 8,000 × *g* to reduce viscosity. Samples were stored at −80°C until protein concentrations were measured and used for SDS-PAGE/western blotting.

#### Cells for flow cytometry

All medium was removed from the cells and the cells were gently washed with PBS prior to lifting the cells off the plates using 5 mM EDTA in PBS. The cells were pelleted at 500 × *g* for 3 min, after which the EDTA/PBS solution was removed, and the cells were then fixed in 4% paraformaldehyde (PFA; Electron Microscopy Services, 15710) in PBS for 15 min at room temperature. The cells were pelleted at 2500 × *g* for 2 min, after which the PFA/PBS solution was removed, and the cells were resuspended in PBS and stored at 4°C prior to staining for flow cytometry.

#### Cells for immunofluorescence

293A cells were seeded onto #1.5 round cover glass (VWR, MARI0117580) and infected as indicated above. All medium was removed from the cells and the cells were gently washed with PBS. The cells were then fixed in PFA in PBS for 15 min at room temperature. The fixation solution was removed, and the cells were stored in PBS at 4°C prior to staining for immunofluorescence.

### RT-qPCR

Total cell RNA was extracted from lysates prepared in Buffer RLT Plus using the RNeasy Plus Mini Kit (QIAGEN) according to the manufacturer’s instructions. Total RNA was converted to cDNA using the Maxima H Minus First Strand cDNA Synthesis Kit (Thermo Fisher, K1652) following the manufacturer’s “RT-qPCR-First Strand cDNA Synthesis” protocol using random hexamers and oligo (dT)_18_ primers with an extended 50°C synthesis step for 30 min. qPCRs were performed using Luna Universal qPCR Master Mix (NEB, M3003X) in 10 µL reactions with 200 nM primers (Table S5) and 1:200 diluted cDNA. One universal forward primer, 5′ UTR (leader; Table S5), was used for all gRNA/sgRNA reactions. qPCRs were performed using CFX Connect Real-Time PCR Detection System (Bio-Rad) using the following two-step cycling conditions: 3 min at 95°C, 40 cycles of 10 s at 95°C, and 30 s at 60°C followed by a 65°C–95°C melt curve. Analysis was performed using an efficiency-corrected 2^−ΔΔCq^ method (90).

### SDS-PAGE and western blotting

Protein concentrations of lysates for SDS-PAGE were determined using the DC Protein Assay (Bio-Rad, 5000116) against a bovine serum albumin (BSA) standard curve and measured in a 96-well plate format at 750 nm using an Eon (BioTek) microplate spectrophotometer. Dithiothreitol (DTT; 108 mM final) and bromophenol blue (BPB; 0.001% final) were added to the protein lysates prior to loading. 5 µg of total protein was loaded into 6% or 12.5% acrylamide gels with Color Prestained Protein Standard, Broad Range (NEB, P7719S) and separated at 100 V. For samples used for PNGase F treatment, 4 µg of each lysate in 2× Laemmli buffer containing DTT/BPB was combined with GlycoBuffer 2 (1× final; NEB, P0704S) and NP-40 (1% final; NEB, P0704S) with or without 1 µL/reaction of PNGase F (NEB, P0704S) and incubated for 1 h at 37°C prior to loading into 12.5% acrylamide gels. Proteins were transferred to polyvinylidene fluoride (PVDF) membranes using the Trans-Blot Turbo RTA Midi 0.2 µm PVDF Transfer Kit (Bio-Rad, 1704273) and a Trans-Blot Turbo Transfer System (Bio-Rad). Membranes were blocked (1 h, room temperature) and probed with primary (overnight, 4°C) and secondary antibodies (1 h, room temperature) in 4% BSA in TBST, except for blots for OC43-HE which were blocked in 4% skim milk in TBST. Primary antibodies used: Mouse anti-coronavirus, OC43 strain (OC43-N; Sigma-Aldrich, MAB9012, 1:2,500); rabbit anti-GFP (mClover3-H2B; Cell Signaling, 2555, 1:1,000); rabbit anti-OC43-S (CUSABIO, CSB-PA336163EA01HIY, 1:2,000); rabbit anti-OC43-HE (M. Desforges, 1:500); and rabbit anti-β-Actin (HRP conjugate; Cell Signaling, 5125, 1:1,000). Secondary antibodies used: Anti-rabbit IgG, HRP-linked (Cell Signaling, 7074, 1:5,000) and anti-mouse IgG, HRP-linked (Cell Signaling, 7076, 1:5,000). All blots were developed using Clarity ECL Western Blotting Substrate (Bio-Rad, 170-5061) and imaged using a ChemiDoc MP Imaging System (Bio-Rad).

### Flow cytometry

After fixation, the cells were pelleted at 2,500 × *g* for 2 min and then permeabilized with 0.1% saponin (Sigma-Aldrich, 47036) in PBS (0.22 µm filtered) for 10 min at 4°C. The cells were stained in 0.1% saponin in PBS with mouse anti-OC43-N (MAB9012, 1:400) for 20 min at 4°C. The cells were washed twice with PBS with centrifugation at 2500 × *g* for 2 min. F(ab')2 goat anti-mouse secondary antibody (Alexa Fluor 647; Thermo Fisher, A-21237, 1:500) was added in 0.1% saponin in PBS and incubated with the cells for 20 min at 4°C. After two washes with PBS (2500 × *g* for 2 min), the cells were resuspended in 1% BSA/1 mM EDTA in PBS (0.22 µm filtered) and stored at 4°C prior to analysis. Samples were analyzed using a CytoFLEX flow cytometer (Beckman Coulter) configured with 405 nm, 488 nm, and 638 nm lasers. Data analysis was performed using FCS Express v6.06.0040 (*De Novo* Software) to determine the percentage of mClover3-H2B and OC43-N (Alexa Fluor 647) positive single cells.

### Cell fluorescence and immunofluorescence microscopy

For imaging reporter protein expression, the cell monolayers in 12-well plates were washed with PBS and fixed with 4% PFA in PBS for 15 min at room temperature. After removing the PFA, the cells were washed with PBS and then visualized in the 12-well plates using an EVOS FL Cell Imaging System (Thermo Fisher) using a 10× objective with DAPI, GFP, RFP, and Cy5 light cubes. For immunofluorescence, cells grown and infected on cover glass were blocked and permeabilized in staining buffer (1% heat-inactivated human serum (Sigma-Aldrich, H3667), 0.1% Triton X-100 in PBS) for 1 h at room temperature, then stained with 1:500 dilution of mouse anti-dsRNA monoclonal antibody J2 (SCICONS, RNT-SCI-10010200) overnight at 4˚C. The following day the cells were washed three times with PBS, then stained with chicken anti-mouse Alexa Fluor 647 (Thermo Fisher, A-21463) in the staining buffer for 1 h at room temperature in the dark. The cells were washed three times with PBS prior to DNA staining with Hoechst 33342 (Thermo Fisher, 62249) for 5 min at room temperature. Following three final washes with PBS, the cover glasses were mounted on slides using ProLong Gold Antifade Mountant (Thermo Fisher, P36930). Z-stacks were imaged on a Zeiss LSM 880 laser scanning confocal microscope with 405 nm, 458/488 nm, 548 nm, and 633 nm lasers using a 63× objective and processed into maximum intensity projections using Zen Black v1.0 (Zeiss).

### Reagents

All oligonucleotides >70 bp in length were PAGE-purified and purchased from Integrated DNA Technologies (IDT). Standard oligonucleotides (<40 bp) were purchased from IDT or Sigma-Aldrich. All restriction enzymes were purchased from NEB except for *Kfl*I (Thermo Fisher, FD2164). All plasmids were propagated in Stbl3 *E. coli* cells and purified using QIAGEN QIAprep Spin Miniprep (27104) or QIAfilter Plasmid Midi (12243) kits according to the manufacturer’s instructions.

### Data management and analysis

All data management was performed with Microsoft Excel for Microsoft 365. Nucleotide and protein sequences were analyzed, and plasmid maps were generated using SnapGene v7.2.1 (Dotmatics). Graphing and statistical calculations were performed using GraphPad Prism for Windows v10.2.3. Figures were prepared using Affinity Designer v1.10.6.1665 (Serif).

## Data Availability

The sequences of the plasmids used to rescue infectious HCoV-OC43 wild-type and reporter viruses are available in GenBank using the following accession numbers: CMVn-OC43-WT-Ribo-BGH-YCpBAC (GenBank PQ791637), CMVn-OC43-mClover-Ribo-BGH-YCpBAC (GenBank PQ791638), CMVn-OC43-mRuby-Ribo-BGH-YCpBAC (GenBank PQ791639), and CMVn-OC43-mCardinal-Ribo-BGH-YCpBAC (GenBank PQ791640).

## SUPPLEMENTAL MATERIAL

Supplemental tables jvi.01671-24-s0001.pdf Tables S1 to S5.

ASM does not own the copyrights to Supplemental Material that may be linked to, or accessed through, an article. The authors have granted ASM a non-exclusive, world-wide license to publish the Supplemental Material files. Please contact the corresponding author directly for reuse.
