# Supplementary material for: A yeast-based reverse genetics system to generate HCoV-OC43 reporter viruses encoding an eighth subgenomic RNA
Source: J Virol. 2025 Jan 30;99(2):e01671-24. doi: 10.1128/jvi.01671-24 (PMC11852775; doi:10.1128/jvi.01671-24)
Supplement: Supplemental tables — Tables S1 to S5. [file jvi.01671-24-s0001.pdf]

Table S1: Oligonucleotides for TAR cloning and screening

Primer	Sequence (5’-3’)
OC43-TAR1F *(T7pro)*	AGAGATCAGTGAAGCGGGATGCACGCACGCAAATCGCTCACAATC***CTATAGTGAGTCGTATTA***GAAGTTATATTCGATGCGGCCGC
OC43-TAR1R	GATTGTCATTGAATTGATAGTTAGTTACGCCCTGTATACGGCATGGCGGCCGCGTATAATGTATGCTATACGAAGTTATTAGCGATGAGCT
OC43-TAR2F	CATGCCGTATACAGGGCGTAACTAACTATCAATTCAATGACAATCATTACCCTGTTATCCCTAGGATCCTCTAGAGTCGACCTGCAG
OC43-TAR2R	GTTGTTTCAATTGGCTACCATTATAAAGAACTTGGTATTGTGATGTAGGGATAACAGGGTAATCCGGGTACCGAGCTCGAATTC
OC43-TAR3F	CATCACAATACCAAGTTCTTTATAATGGTAGCCAATTGAAACAACATTACCCTGTTATCCCTAGGATCCTCTAGAGTCGACCTGCAG
OC43-TAR3R	AGAAGTTTTCGTTGGTGACAGTTTGGTTAATGTAATCTAAACTTTTAGGGATAACAGGGTAATCCGGGTACCGAGCTCGAATTC
OC43-TAR4F	AAAGTTTAGATTACATTAACCAAACTGTCACCAACGAAAACTTCTATTACCCTGTTATCCCTAGGATCCTCTAGAGTCGACCTGCAG
OC43-TAR4R	GGCATGGACACCGCATTGTTGAGAAATAATATCTAAATTTTAAGGTAGGGATAACAGGGTAATCCGGGTACCGAGCTCGAATTC
OC43-TAR5F-WT	TACGTCTCGCGTGGTTTTCTTGACAGGATAAG
OC43-TAR5R-WT	TACGTCTCTGGGTTGAGCTCTTCTACCCC
OC43-TAR5F-mClover	TAGCGAATTCATTACCCTGTTATCCCTAGGCATGGACACCGCATTGTTGAGAAATAATATCTAAATTTTAAGGGTTTAGTGAACCGTCAGATCCGCTAGC
OC43-TAR5R-mClover	TAGCAAGCTTTAGGGATAACAGGGTAATTCTACTACTGGATTGCTTACCAGGAGTAAAAGACATCCTTAAAATGTTTAAACCTCTACAAATGTGGTATGGCTGATTATG
AgeI-TAR5F-FLuc	AGCGCTACCGGTCGCCACCATGGAAGACGCCAAAAAC
BamHI-TAR5R-FLuc	CGGTGGATCCCTTACTCGAGTTTGGACTTTCCG
OC43-TAR6F	TCTACTACTGGATTGCTTACCAGGAGTAAAAGACATCCTTAAAATATTACCCTGTTATCCCTAGGATCCTCTAGAGTCGACCTGCAG
OC43-TAR6R (***poly(A)***)	ATTAGTAAATGAATGAAGTTAATTATGGCCAATTGGAAGAATCAC***AAAAAAAAAAAAAAAAAAAAAAAAAAAAAAAAAA***TAGGGATAACAGGGTAATCCGGGTACCGAGCTCGAATTC
OC43-TAR7F (***poly(A)***) (YCpBAC)	CAATTGGAAGAATCAC***AAAAAAAAAAAAAAAAAAAAAAAAAAAAAAAAAA***GAAGTTATATTCGATGCGGCCGC
OC43-TAR7R (***T7pro***) (YCpBAC)	GCGGGATGCACGCACGCAAATCGCTCACAATC***CTATAGTGAGTCGTATTA***GCGGCCGCGTATAATGTATGCTATACGAAGTTATTAGCGATGAGCT
OC43-TAR7F-trunc (YCpBAC)	CGACACTGAATACGGGGCAAC
OC43-TAR7R-trunc (YCpBAC)	TACAGCCAGTAGTGCTCGCC
7/1F	ACAACAGTACTGCGATGAGTGG
7/1R	TCTACGCCCACAAGCATAGATTAC
1/2F	GAGAGCATTTGTTGATTATACAGGTGTG
1/2R	GATTTACTACAACCATAGGCAACATGC
2/3F	ACCATGTGTCATGCATTGGATTG
2/3R	CATGCAAAGCTGGATCAGCAG
3/4F	GCCGTAATAAATTTAAGAGCAGACCAG
3/4R	CTGGATTGGTCAGCAACACAAC
4/5F	ACTAGGTACTGGCTATTCTTTGGC
4/5R	GTAGCGCTAGCGGATCTG
4/6R	ATACCATCGTGGCAGCAGTT
5/6F	TGTACAAGTCCGGACTCAGATC
5/6F-FLuc	CAAGAAGGGCGGAAAGTCC
5/6R	CCTGATGGTTGCTGAGAGGTAG
6/7F	GCTATAACGGCGCAATTAGGTTTG

6/7R	ACAATGGAAGTCCGAGCTCATC
TAR1ampF	GATTGTGAGCGATTTGCGTGC
TAR6ampR	TTTGTGATTCTTCCAATTGGCCATAATTAAC
RCO495.F (49)	ACGACGGCCAGTGAATTG
BACYCp.R	CGCCAAGCTATTTAGGTGAGAC
Ribo_R	GCTGTCGACGAGCTCG
BD205	CCAGCATGTTAAGTTACCACCCAG
BD206	TTAATTAACTGTGCCTTCTAGTTGCCAGC
BD207	ATTACCCTGTTATCCCTACCATAGAGCCCACCGCATCC
CMVn-OC43F	CGCAAATGGGCGGTAGGCGTGTACGGTGGGAGGTCTATATAAGCAGAGCTCGTTTAGTGA ACCGT
CMVn-OC43R	AAGATCTAACAAGAGATCAGTGAAGCGGGATGCACGCACGCAAATCGCTCACAATACGGT TCACTAAA

Table S2: Screening amplicon sizes following YCpBAC assembly

Amplicon (primer pair)	Size (bp)	Amplicon (primer pair)	Size (bp)
OC43-TAR1 5’ (7/1F+7/1R)	321	OC43-TAR1 3’ (1/2F+6/7R)	138
OC43-TAR2 5’ (BACYcp.R+1/2R)	349	OC43-TAR2 3’ (2/3F+ RCO495.F)	396
OC43-TAR3 5’ (BACYcp.R+2/3R)	212	OC43-TAR3 3’ (3/4F+ RCO495.F)	212
OC43-TAR4 5’ (BACYcp.R+3/4R)	508	OC43-TAR4 3’ (4/5F+ RCO495.F)	301
OC43-TAR6 5’ (BACYcp.R+5/6R)	264	OC43-TAR6 3’ (6/7F+ RCO495.F)	693
7/1 (*T7pro*) (7/1F+7/1R)	321	7/1 (*CMVpro*) (7/1F+7/1R)	879
1/2 (1/2F+1/2R)	294	2/3 (2/3F+2/3R)	397
3/4 (3/4F+3/4R)	509	3/7 (3/4F+6/7R)	571
4/5 (4/5F+4/5R)	249	4/6 (4/5F+4/6R)	595
5/6 (mClover) (5/6F+5/6R)	660	5/6 (mRuby) (5/6F+5/6R)	660
5/6 (mCardinal) (5/6F+5/6R)	312	5/6 (EBFP) (5/6F+5/6R)	312
5F/6 (FLuc) (5/6F-FLuc+5/6R)	276	6/7 (*-Ribo*) (6/7F+ RCO495.F)	693
6/7* (+*Ribo*) (6/7F+6/7R)	1207	7/4 (BACYcp.R+3/4R)	508

Table S3: Single-nucleotide polymorphisms in YCpBACs used for virus rescue compared to a reference stain of HCoV-OC43

Plasmid	Nucleotide change	Affected gene	Type of amino acid substitution
WT/EBFP/mClover/mRuby/mCardinal	28U>C	5'UTR	N/A
WT/EBFP/mClover/mRuby/mCardinal	1237U>C	*Orf1a*	Missense, V344A
WT/EBFP/mClover/mRuby/mCardinal	1898C>U	*Orf1a*	Synonymous, A564
WT/EBFP/mClover/mRuby/mCardinal	2162C>U	*Orf1a*	Synonymous, F652
EBFP	4683G>U	*Orf1a*	Missense, A1493S
WT/EBFP/mClover/mRuby/mCardinal	4694C>U	*Orf1a*	Synonymous, F1496
WT/EBFP/mClover/mRuby/mCardinal	5256U>C	*Orf1a*	Missense, Y1684H
EBFP	5315C>A	*Orf1a*	Synonymous, R1703
WT/EBFP/mClover/mRuby/mCardinal	6415C>U	*Orf1a*	Missense, S2070F
WT/mCardinal	9149U>C	*Orf1a*	Synonymous, G2981
WT/EBFP/mClover/mRuby/mCardinal	9155C>U	*Orf1a*	Synonymous, C2983
WT/mCardinal	11190G>U	*Orf1a*	Missense, V3662F
WT/EBFP/mClover/mRuby/mCardinal	12311C>U	*Orf1a*	Synonymous, A4035
mCardinal	12680G>A	*Orf1a*	Synonymous, G4158
WT/EBFP/mClover/mRuby/mCardinal	14583U>C	*Orf1b*	Missense, P4793L
WT/EBFP/mClover/mRuby/mCardinal	24155G>U	*S*	Missense, E172D
WT/EBFP/mClover/mRuby/mCardinal	24387U>C	*S*	Missense, Y250H
EBFP	25595U>A	*S*	Missense, N652K
WT/EBFP/mClover/mRuby/mCardinal	278645_27872del	*NS5A*	Deletion, I14_G16del
WT/EBFP/mClover/mRuby/mCardinal	27947U>C	*NS5A*	Synonymous, V41
WT/EBFP/mClover/mRuby/mCardinal	28982C>U	*M*	Synonymous, D191
WT	29282G>A	*N*	Synonymous, G57
WT/EBFP/mClover/mRuby/mCardinal	29289G>A	*N*	Missense, V60I
WT/EBFP/mClover/mRuby/mCardinal	30068A>C	*N*	Synonymous, T319
EBFP	30465G>U	*3'UTR*	N/A

Comparison to HCoV-OC43 VR-1558 (GenBank accession# ON376724). Nucleotide and amino acid numbering are relative to ON376724. Abbreviations: EBFP, CMVn-OC43-EBFP-Ribo-BGH-YCpBAC; *M*, membrane; mCardinal, CMVn-OC43-mCardinal-Ribo-BGH-YCpBAC; mClover, CMVn-OC43-mClover-Ribo-BGH-YCpBAC; mRuby, CMVn-OC43-mRuby-Ribo-BGH-YCpBAC; *N*, nucleocapsid; *NS5A*, nonstructural gene 5A; *Orf1a*, open reading frame 1a; *S*, spike; UTR, untranslated region; WT, CMVn-OC43-WT-Ribo-BGH-YCpBAC.

Table S4: Plasmids generated for HCoV-OC43 TAR assembly

Name	Description
OC43TAR1-YCpBAC	TAR cloning plasmid containing a 7178 bp fragment of OC43 cDNA sequence from the 5' UTR to residue 1471 of *Nsp3*. The 5'UTR is cloned immediately downstream of a T7 promoter sequence.
OC43TAR2-YCpBAC	TAR cloning plasmid containing a 7236 bp fragment of OC43 cDNA sequence from residue 1458 of *Nsp3* to residue 351 of *Nsp12*.
OC43TAR3-YCpBAC	TAR cloning plasmid containing a 7179 bp fragment of OC43 cDNA sequence from residue 337 of *Nsp12* to *Nsp16*.
OC43TAR4-YCpBAC	TAR cloning plasmid containing a 7646 bp fragment of OC43 cDNA sequence from *NS2A* to *M*.
pUC57-N-TRS	Plasmid containing a synthesized *OC43-N* TRS for insertion by TAR cloning downstream of reporter gene sequences and upstream of the *OC43-N* start codon.
pcDNA3.1-OC43TAR5-mClover3-H2B-TRS	Cloning plasmid containing the *mClover3-H2B* gene followed by a synthetic *N* TRS flanked by HCoV-OC43 sequences to introduce the reporter gene between *M* and *N*.
pcDNA3.1-OC43TAR5-mRuby3-H2B-TRS	Cloning plasmid containing the *mRuby3-H2B* gene followed by a synthetic *N* TRS flanked by HCoV-OC43 sequences to introduce the reporter gene between *M* and *N*.
pcDNA3.1-OC43TAR5-mCardinal-TRS	Cloning plasmid containing the *mCardinal* gene followed by a synthetic *N* TRS flanked by HCoV-OC43 sequences to introduce the reporter gene between *M* and *N*.
pcDNA3.1-OC43TAR5-EBFP-TRS	Cloning plasmid containing the *EBFP2* gene followed by a synthetic *N* TRS flanked by HCoV-OC43 sequences to introduce the reporter gene between *M* and *N*.
pcDNA3.1-OC43TAR5-FLuc-TRS	Cloning plasmid containing the firefly *luciferase* gene followed by a synthetic *N* TRS flanked by HCoV-OC43 sequences to introduce the reporter gene between *M* and *N*.
OC43TAR6-YCpBAC	TAR cloning plasmid containing a 1644 bp fragment of OC43 cDNA sequence from *N* to the 3' UTR. An encoded $A_{(34)}$ sequence is immediately downstream of the 3' UTR.
OC43TAR123-YCpBAC	TAR cloning plasmid containing a 21502 bp fragment of OC43 cDNA sequence encoding the 5' UTR and *Orf1ab*. The 5'UTR is cloned immediately downstream of a T7 promoter sequence.
OC43TAR456-WT-YCpBAC	TAR cloning plasmid containing a 9315 bp fragment of OC43 cDNA sequence encoding the last 12 residues of *Orf1b*, all genes from *NS2A* to *N*, the 3'UTR, and a synthetic poly(A) sequence.
OC43TAR456-mClover-YCpBAC	Similar to OC43TAR456-WT except containing the *mClover3-H2B* gene and a synthetic TRS inserted between *M* and *N*.
OC43TAR456-mRuby-YCpBAC	Similar to OC43TAR456-WT except containing the *mRuby3-H2B* gene and a synthetic TRS inserted between *M* and *N*.
OC43TAR456-mCardinal-YCpBAC	Similar to OC43TAR456-WT except containing the *mCardinal-H2B* gene and a synthetic TRS inserted between *M* and *N*.
OC43TAR456-EBFP-YCpBAC	Similar to OC43TAR456-WT except containing the *EBFP2* gene and a synthetic TRS inserted between *M* and *N*.
OC43TAR456-FLuc-YCpBAC	Similar to OC43TAR456-WT except containing the firefly *luciferase* gene and a synthetic TRS inserted between *M* and *N*.
OC43-mClover-YCpBAC	Plasmid encoding the OC43-$mClo^{YA}$ reporter virus for rescue using *in vitro* T7 RNA pol transcription from a *I-Sce*-I-linearized template.
OC43-mClover-Ribo-YCpBAC	Plasmid encoding the OC43-$mClo^{YA}$ reporter virus with HDV ribozyme for rescue using *in vitro* T7 RNA pol transcription from a *Not*-I-linearized template.
OC43-WT-Ribo-YCpBAC	Plasmid encoding the $OC43^{YA}$ virus with HDV ribozyme for rescue using *in vitro* T7 RNA pol transcription from a *Not*-I-linearized template.
pUC57-HDV-T7term	Plasmid containing synthesized HDV ribozyme/T7 terminator sequences flanked by OC43-3'UTR/YCpBAC homology for TAR cloning.
pcDNA3.1-CMV-OC43-5'UTR	Contains CMV enhancer/CMV promoter/T7 promoter sequences flanked by YCpBAC/OC43-5'UTR homology for TAR cloning.
pUC19pc	Intermediate pUC19 cloning vector containing a pcDNA3.1+ MCS.
pUC19pc-cos-BGH	Contains the BGH poly(A) signal inserted with flanking YCpBAC homology for TAR cloning into YCpBACs containing the HDV ribozyme/T7 terminator sequences.
CMV-OC43-mClover-Ribo-BGH-YCpBAC	Plasmid encoding the OC43-$mClo^{YA}$ reporter virus with HDV ribozyme for rescue *in vivo* using a CMV promoter and BGH poly(A) signal. Contains the T7 promoter sequence downstream of the CMV promoter.

CMV-OC43-WT-Ribo-BGH-YCpBAC	Plasmid encoding the $OC43^{YA}$ virus with HDV ribozyme for rescue *in vivo* using a CMV promoter and BGH poly(A) signal. Contains the T7 promoter sequence downstream of the CMV promoter.
CMVn-OC43-mClover-Ribo-BGH-YCpBAC	Plasmid encoding the $OC43\text{-}mClo^{YA}$ reporter virus with HDV ribozyme for rescue *in vivo* using a CMV promoter and BGH poly(A) signal. This plasmid lacks the T7 promoter sequence and uses CMV promoter/OC43 5'UTR spacing used by St-Jean *et al*. (18).
CMVn-OC43-WT-Ribo-BGH-YCpBAC	Plasmid encoding the $OC43^{YA}$ virus with HDV ribozyme for rescue *in vivo* using a CMV promoter and BGH poly(A) signal. This plasmid lacks the T7 promoter sequence and uses CMV promoter/OC43 5'UTR spacing used by St-Jean *et al*. (18).
CMVn-OC43-mRuby-Ribo-BGH-YCpBAC	Plasmid encoding the $OC43\text{-}mRuby^{YA}$ reporter virus with HDV ribozyme for rescue *in vivo* using a CMV promoter and BGH poly(A) signal. This plasmid lacks the T7 promoter sequence and uses CMV promoter/OC43 5'UTR spacing used by St-Jean *et al*. (18).
CMVn-OC43-mCardinal-Ribo-BGH-YCpBAC	Plasmid encoding the $OC43\text{-}mCard^{YA}$ reporter virus with HDV ribozyme for rescue *in vivo* using a CMV promoter and BGH poly(A) signal. This plasmid lacks the T7 promoter sequence and uses CMV promoter/OC43 5'UTR spacing used by St-Jean *et al*. (18).
CMVn-OC43-EBFP-Ribo-BGH-YCpBAC	Plasmid encoding the $OC43\text{-}EBFP^{YA}$ reporter virus with HDV ribozyme for rescue *in vivo* using a CMV promoter and BGH poly(A) signal. This plasmid lacks the T7 promoter sequence and uses CMV promoter/OC43 5'UTR spacing used by St-Jean *et al*. (18).

Table S5: qPCR primers

Name	Direction	Sequence (5’-3’)
5’UTR (leader)	F	CCGCTTCACTGATCTCTTG
Orf1a	R	ACCACTATGAAAAATCTACGCC
sg*NS2A*	R	GGAAAATTGATAAAATGATTAGGCTTGTC
sg*HE*	R	ATGCAGCTAACTAGAATAAATCTAGG
sg*S*	R	AGTACACTTTAAATCTCCTATAACAGC
sg*NS5A*	R	CTAACAAGAAATCAGTGCAATGAATC
sg*E*	R	ACCAGTTTGTTAACAGCATCC
sg*M*	R	GTAGTATAATACCCAAAGAAAAATTCCATTC
sg*mClover*	R	CCCTTAGACACCATGGTGG
sg*N*	R	GAGGACGCTCTACTACTGG
